# Analysis of Copy-Number Variations and Feline Mammary Carcinoma Survival

**DOI:** 10.1038/s41598-020-57942-7

**Published:** 2020-01-22

**Authors:** José Luis Granados-Soler, Kirsten Bornemann-Kolatzki, Julia Beck, Bertram Brenig, Ekkehard Schütz, Daniela Betz, Johannes Junginger, Marion Hewicker-Trautwein, Hugo Murua Escobar, Ingo Nolte

**Affiliations:** 1 Small Animal Clinic, University of Veterinary Medicine Hannover Foundation, Hannover, Germany; 2 Chronix Biomedical, Göttingen, Germany; 3 Institute of Veterinary Medicine, University of Göttingen, Göttingen, Germany; 4 Department of Pathology, University of Veterinary Medicine Hannover Foundation, Hannover, Germany; 5 Haematology, Oncology and Palliative Medicine, Clinic III, University of Rostock, Rostock, Germany

**Keywords:** Breast cancer, Translational research, Molecular medicine, Breast cancer

## Abstract

Feline mammary carcinomas (FMCs) are highly malignant. As the disease-free survival (DFS) and overall survival (OS) are short, prognostication is crucial. Copy-number variations (CNVs) analysis by next-generation sequencing serves to identify critical cancer-related genomic regions. Thirty-three female cats with FMCs were followed during two years after surgery. Tumours represented tubulopapillary and solid carcinomas encompassing six molecular subtypes. Regardless of the histopathological diagnosis, molecular subtypes showed important differences in survival. Luminal A tumours exhibited the highest DFS (*p* = 0.002) and cancer-specific OS (*p* = 0.001), and the lowest amount of CNVs (*p* = 0.0001). In contrast, basal-like triple-negative FMCs had the worst outcome (DFS, *p* < 0.0001; and OS, *p* < 0.00001) and were the most aberrant (*p* = 0.05). In the multivariate analysis, copy-number losses (CNLs) in chromosome B1 (1–23 Mb) harbouring several tumour-repressors (e.g. *CSMD1*, *MTUS1*, *MSR1*, *DBC2*, and *TUSC3*) negatively influenced DFS. Whereas, copy-number gains (CNGs) in B4 (1–29 Mb) and F2 (64–82.3 Mb) comprising epithelial to mesenchymal transition genes and metastasis-promoting transcription factors (e.g. *GATA3*, *VIM*, *ZEB1*, and *MYC*) negatively influenced DFS and cancer-specific OS. These data evidence an association between specific CNVs in chromosomes B1, B4 and F2, and poor prognosis in FMCs.

## Introduction

FMCs are generally malignant hormone-independent adenocarcinomas, animals affected have a reduced survival due to early metastatic spreading^1^. The median OS in untreated cats after tumour detection varies from four months to three years depending on tumour size and clinical staging^2–6^. Thus, early diagnosis, prognostication, and appropriate treatment selection are of major importance. In the clinical practice, histological classification^7^ and clinical staging^8^ are currently the most widely used methods to guide treatment. However, according to a literature review by Zappulli *et al*. the most reliable prognostic parameters for FMCs are the histological malignancy grade, lymph node status, and lymphovascular invasion at diagnosis^1^. Considering the lack of protocols standardisation, molecular tool cross-reactivity and consensus on data analysis^1^, further studies in FMCs are still necessary to evaluate the prognostic value of molecular markers (e.g. HER2, ER, PR, and Ki-67) commonly used in human breast cancer (HBC). Recent studies demonstrated the suitability and prognostic value of the HBC immunohistochemistry (IHC)-based St. Gallen molecular classification in FMCs^9,10^. This classification is based on the expression of different molecular markers (i.e. ER, PR, feline-homologue HER2 [*f*HER2], Ki-67 and CK5/6). Accordingly, FMCs are classified into six molecular subtypes exhibiting different biological behaviour and prognosis, including Luminal A (LA), Luminal B *f*HER2-negative (LB *f*HER2−), luminal B *f*HER2-positive (LB *f*HER2+), *f*HER2 positive (*f*HER2+), normal-like triple-negative (normal-like TN), and basal-like triple-negative (basal-like TN). Among those, LA tumours have the best prognosis while the TN subtypes exhibit the worse outcome and poorest survival rates in both humans^11–13^ and cats^10^.

Genomic analysis can provide further prognostic relevant information and may help to understand the molecular pathogenesis of the neoplastic change across different molecular subtypes. In recent years, there has been exponential progress in molecular analysis with profound implications for our understanding of mammary cancer biology^14^. Thereby somatically acquired copy-number variations (CNVs) also known as copy-number alterations (CNAs)—comprising copy-number gains (CNGs) and copy-number losses (CNLs)— have been used to identify and characterise complex genomic rearrangements derived from aneuploidy evolution and chromosomal instability during tumourigenesis and tumour progression^15–18^. In fact, comparative genome hybridisations and mass-parallel sequencing CNVs analyses have been successfully used in HBC research and diagnostics^19–22^. Therefore, CNVs analysis directly contributes to understanding global gene expression deregulation as well as genomic rearrangements with transforming potential in FMCs development and progression^23–25^. Furthermore, specific CNVs can be used to identify genomic regions commonly affected in patients with poor prognosis^23,26^ and poor response to therapy^26–28^ contributing to an individual characterisation of specific cancer subtypes^29,30^. To the best of our knowledge, no studies in FMCs have correlated the frequency and localisation of these structural aberrations with survival. This study explores the influence of CNVs in FMCs survival and its correlation with histopathological diagnosis and molecular subtype.

## Results

### Animals

Thirty-three female cats were included in the study; thirty-one were retrospectively included and two were prospectively enrolled. The age at diagnosis ranged from 6 to 20 years (mean [SD]: 12.4 [3.0] years). Sixteen (48.5%) cats were spayed, the remaining 17 (51.5%) were intact at diagnosis but were spayed at the time of mastectomy. Most of the tumours occurred in the abdominal and inguinal mammary glands (11 [33.3%], and 13 [39.4%] cases; respectively). In six cases (18.2%), tumours affected the axillary mammary glands. The remaining three cats had tumours in the thoracic mammary glands. At diagnosis, 15 cats (45.5%) showed lymph-node metastasis (inguinal or axillary) as confirmed by histopathology. None of the animals included revealed distant metastases at the time point of diagnosis. Histopathologically, twenty-five cats (75.8%) had tubulopapillary carcinomas among which four cats had intraductal papillary carcinomas (group TC). Eight cats (24.2%) showed solid carcinomas (n = 4) or comedocarcinomas (n = 4), both grouped as SC. Additional epidemiological and clinical features of cats included in this study are displayed in Supplementary Table 1.

### Histopathology, immunohistochemistry, and HER2-status validation

Immunohistochemically, ten cases (30.3%) were classified as ER-positive, and 11 as PR-positive (33.3%). Among them, four cases (12.1%) expressed ER and PR simultaneously, all cases positive to ER and/or PR were allocated in group TC. In contrast, all tumours included in the group SC were ER- and PR-negative, Supplementary Table 2. Twenty-six cases (78.8%; TC = 19, and SC = 7) were negative to *f*HER2 (IHC-0 or IHC-1+), five (15.1%; TC = 4, and SC = 1) had an equivocal expression (IHC-2+), and two (both TC) were positive (IHC-3+), Supplementary Table 2. As reported elsewhere^31–33^, we correlated *f*HER2 IHC-based expression with CNGs affecting *HER2* gene-coding region (FCA E1 40,780,250–40,804,241 bp) to validate IHC-2+ cases. We identified a six million base pairs CNG (FCA E1 38–44 Mb) encompassing *HER2* in seven cases (log2-ratio >0.2), four of them with a log2-ratio >0.5 including two, IHC-1+; one, IHC-0; and one, IHC-2+. Accordingly, one case scored as IHC-2+ with *HER2*-associated CNGs was considered as *f*HER2+ for the St. Gallen classification. Moreover, nine cases (27.3%; TC = 6, and SC = 3) were positive for CK5/6, details in Supplementary Table 2.

In group TC, the Ki-67 proliferation index ranged from 2% to 54.2% (mean [SD]: 19.1 [14.4] %); 14 tumours displayed high Ki-67 indexes (≥14%, cut-off value according to Soares *et al*.^34^), while the remaining 11 showed low Ki-67 scores (<14%). On the other hand, all cases included in the group SC were characterised by high Ki-67 indexes (≥14%), ranging from 14.4% to 44.3% (mean [SD]: 26.4 [10.6] %), Supplementary Table 2.

Important differences were observed when applying the three histological grading systems reported on FMCs (i.e. Elston and Ellis [EE]^35,36^, mitotic-modified EE [MMEE]^37,38^, and the novel histological malignancy grading system for evaluation of FMCs [Mills-2015]^37,38^), Supplementary Table 3. There was a minimal level of agreement when the histological grade assigned to each tumour using the three different systems was compared using Cohen's Kappa test: EE and MMEE (K = 0.25), EE and Mills-2015 (K = 0.27), MMEE and Mills-2015 (K = 0.24). Moreover, in only ten out of the 33 cases evaluated (30.3%) tumours obtained the same grade (five, grade I; two, grade II; and three, grade III) when using any of the three grading systems evaluated.

After percent agreement calculation, a moderate level of agreement between well-differentiated tumours (grade I) and low Ki-67 index (<14%) was observed when using EE and Mills-2015 grading systems, as seven out of 12 (58%), and four out of six (66.7%) cases graded as I (EE and Mills-2015, respectively) had low Ki-67 indexes. In contrast, applying the MMEE system, ten out of the 21 tumours (47.6%) graded as I had low Ki-67 indexes. On the other hand, a very strong level of agreement was observed for all three grading systems when correlating poorly-differentiated tumours (grade III) and high Ki-67 indexes (≥14%); EE (9/10, 90%), MMEE (3/3, 100%), and Mills-2015 (11/11, 100%). Using the EE and MMEE systems, moderately-differentiated tumours (grade II) were predominantly scored as high Ki-67 index (9/10, 90%; and 8/9, 88.9%; respectively). On the other hand, nine out of the 16 tumours (56.2%) graded as II using the Mills-2015 system had high Ki-67 indexes and the remaining seven displayed low Ki-67 scores, Supplementary Table 3.

### Molecular subtyping

**Figure 1:**
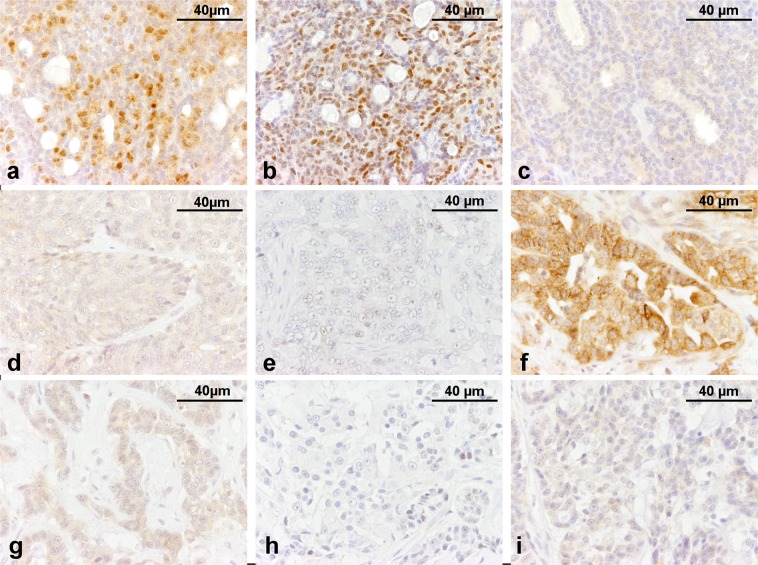
Hormonal status of immunohistochemical-based subtypified FMCs. Luminal A tubular carcinoma (**a**) ER, positive; (**b**) PR, positive; and (**c**) *f*HER2, negative. *f*HER2-positive solid carcinoma (**d**) ER, negative; (**e**) PR, negative; and (**c**) *f*HER2, equivocal (positive expression confirm by increased number of copies in *HER2* genomic region). Basal-like TN tubular carcinoma (**d**) ER, negative; (**e**) PR, negative; and (**f**) *f*HER2, negative.

The LA molecular subtype (Fig. 1a–c) was the most common, with a frequency of 30.3% (10/33), followed by the normal-like TN subtype 24.2% (8/33). Two cases (*f*HER2 IHC-3+) were classified as LB *f*HER2+. After confirmation of CNGs encompassing *HER2*, one case (*f*HER2 IHC-2+) was confirmed as *f*HER2+ subtype (Fig. 1d–f). LB *f*HER2−, and basal-like TN (Fig. 1g–i) tumours were detected in ten cases, five cases (15.1%) each subtype. Moreover, in two cases included in the TC group subtyping was not performed due to loss of the neoplastic lesion from the paraffin block after consecutive slicing, in those cases, it was not possible to evaluate all required markers to assess the St. Gallen classification.

Tumours in the group TC (n = 25) included multiple molecular subtypes. In this group, ten cats (40%) had LA subtype tumours. Whereas four cats (16%) had normal-like TN tumours, five cats (20%) had LB *f*HER2− tumours, two cats had LB *f*HER2+ tumours, two cats had basal-like TN tumours, and two cats were not classified, Supplementary Table 2. Interestingly, LA subtype tumours (all included in group TC) had a significantly lower Ki-67 index (*p* < 0.001) than any other molecular subtype. The group SC (n = 8) was less heterogeneous in terms of molecular diversity. In this group, seven cats (87.5%) had triple-negative FMCs including four normal-like TN and three basal-like TN tumours. Moreover, one cat included in this group was classified as *f*HER2+, Supplementary Table 2.

### Tissue samples DNA quantification

Thirty-three tumours were included: 23 formalin-fixed paraffin-embedded (FFPE) samples (group TC = 16, and group SC = 7), and ten fresh-frozen (FT) samples (group TC = 9; and group SC = 1). The DNA yields from FT samples were more consistent when compared to those of FFPE samples, ranging from 1466.2 to 16538.7 ng (mean [SD]: 7727.8 [4915.7] ng). In contrast, DNA yields from FFPE samples varied greatly and included some outliers, DNA values ranged from 2406.8 to 52201.7 ng (mean [SD]: 16746.7 [12991.5] ng). A 260/280 ratio of ~1.8 was observed in all samples evaluated.

In the group TC, four FFPE samples stored for over 15 years (LA = 1, LB *f*HER2− = 1, normal-like TN = 1, and basal-like TN = 1) failed library preparation due to low DNA quality. In three (two FFPE, and one FF) samples (all LA subtype) of the same group, no CNVs were detected, these seven samples were later excluded from CNVs analysis. Twenty-six samples were suitable for CNVs analysis (FFPE = 17, and FT = 9) as follows: LA = 6, LB *f*HER2+ = 2, LB *f*HER2− = 4, *f*HER2+ = 1, normal-like TN = 7, basal-like TN = 4, and not-classified = 2; distribution of molecular subtypes across histological groups (TC and SC) is detailed in Supplementary Table 2.

### Follow-up and survival analysis censoring

At the end of the study period (24 months), 12 cats (48%) in the group TC (n = 25) had developed local recurrence and eight of them (32%) subsequent distant metastasis (eight, pulmonary/pleural; and one, intestinal). One cat (normal-like TN) in this group developed pulmonary/pleural metastasis without previous local recurrence, and 12 cats (48%) did not develop any kind of recurrence (Supplementary Table 4); cats without recurrence (n = 12) at the end of the study period were censored from DFS analysis.

Eight cats (32%) in the group TC (LA = 7, and not-classified = 1) were still alive at the time of censorship; however, two of them developed local recurrence (21, and 24 months after surgery). In 11 cases (44%) included in group TC (44%) the cause of death was related to the progression of the neoplastic disease, while six cats (24%) died due to non-tumour related causes as follows: two, anaesthetic complications during mastectomy; two, renal failure; one, euthanised during mastectomy due to concomitant intestinal primary tumour and liver metastasis; and one, severe cardiac disease (Supplementary Table 4). All animals alive at the end of the study period (n = 8) and those who died due to non-tumour related causes (n = 6) were censored from cancer-specific OS analysis.

In the group SC (n = 8), five cats (63%) developed local recurrence and pulmonary/pleural metastasis, and three cats (all basal-like TN) developed pulmonary/pleural metastasis without previous local recurrence, Supplementary Table 4. Additionally, all animals in group SC died due to the progression of the neoplastic disease. Accordingly, none of the animals in the group SC was censored from DFS and cancer-specific OS analysis. After censorship, 21 cats (TC = 13, and SC = 8) were included in the DFS analysis and 19 cats (TC = 11, and SC = 8) were included in the cancer-specific OS analysis.

### CNVs detection and frequency analysis

140 CNVs were detected using CNV-seq^39^, and 99 using CNVKit^40^ including 97 overlapped regions (69%) of the 140 CNVs detected with CNV-seq. Details on number of mapped reads per sample, and aberrant genomic windows detected using each CNV caller independently (CNV-seq and CNVKit) are provided in Supplementary Table 5. Among CNVs identified with both callers, 97 overlapped genomic regions affected by CNVs were delimited between both algorithms (CNV-seq and CNVKit), Supplementary Table 6. Validated and refined CNVs (n = 97, mean [SD]: 23.1 [18.1] Mb) comprised 29 regions exclusively affected by CNLs, 22 by CNGs, and 46 affected by CNGs or CNLs in different patients, Supplementary Table 6. Among validated regions, 27 CNVs (CNGs = 12, and CNLs = 15) were detected in more than 20% of the cases in which CNVs analysis was performed (n = 26), nine of them were observed in more than 30% of the patients, and only one (CNG in FCA E3 1.1–34.5 Mb) in more than 40% of the patients, Supplementary Table 6.

### CNVs scores across histological subtypes

The percentage of overlapped aberrant genomic windows detected with both CNV callers ranged from 0.2 to 68.6% (n = 26, mean [SD]: 17.6 [19.2] %). In the group SC (n = 8), the amount of aberrant bins (4.05 to 68.6%; mean [SD]: 29.5 [24.2] %) was significantly higher (*p* = 0.03) than that of the group TC (n = 18; 0.22 to 51.6%; mean [SD]: 12.3 [15.03] %). Accordingly, eleven samples in the group TC (n = 18) were scored as low-CNVs (<median overlapped aberrant genomic windows percentage), while the remaining seven as high-CNVs (≥median overlapped aberrant genomic windows). In contrast, group SC included six samples with high-CNVs and two with low-CNVs, Supplementary Table 5.

In both groups (TC and SC) CNVs affected all chromosomes. However, the frequency of CNVs in the group TC was very low in comparison to the group SC. In group TC (n = 18), 81 among the 97 validated CNVs were observed in less than 20% (4/18) of the cases, 13 were not detected in any of the patients allocated in the group, and only one (CNG in FCA E3 37–42.5 Mb) was observed in at least 30% (6/18) of the cats included in the group, Supplementary Table 6. In contrast, group SC displayed a higher frequency of CNVs, 71 among the 97 validated CNVs were detected in more than 20% (2/8) of the patients allocated in group SC, 24 CNVs were observed in at least 50% (4/8), and one (CNG in FCA B4 1–29 Mb) was observed in 75% (6/8) of the cases, Supplementary Table 6. On the other hand, poorly differentiated tumours (grade III, according to the EE system), and tumours with high Ki-67 index showed a significantly higher amount of aberrant genomic windows affected by CNVs (*p* = 0.01, and *p* = 0.0002; respectively).

### Survival differences among histological groups and molecular subtypes

**Figure 2:**
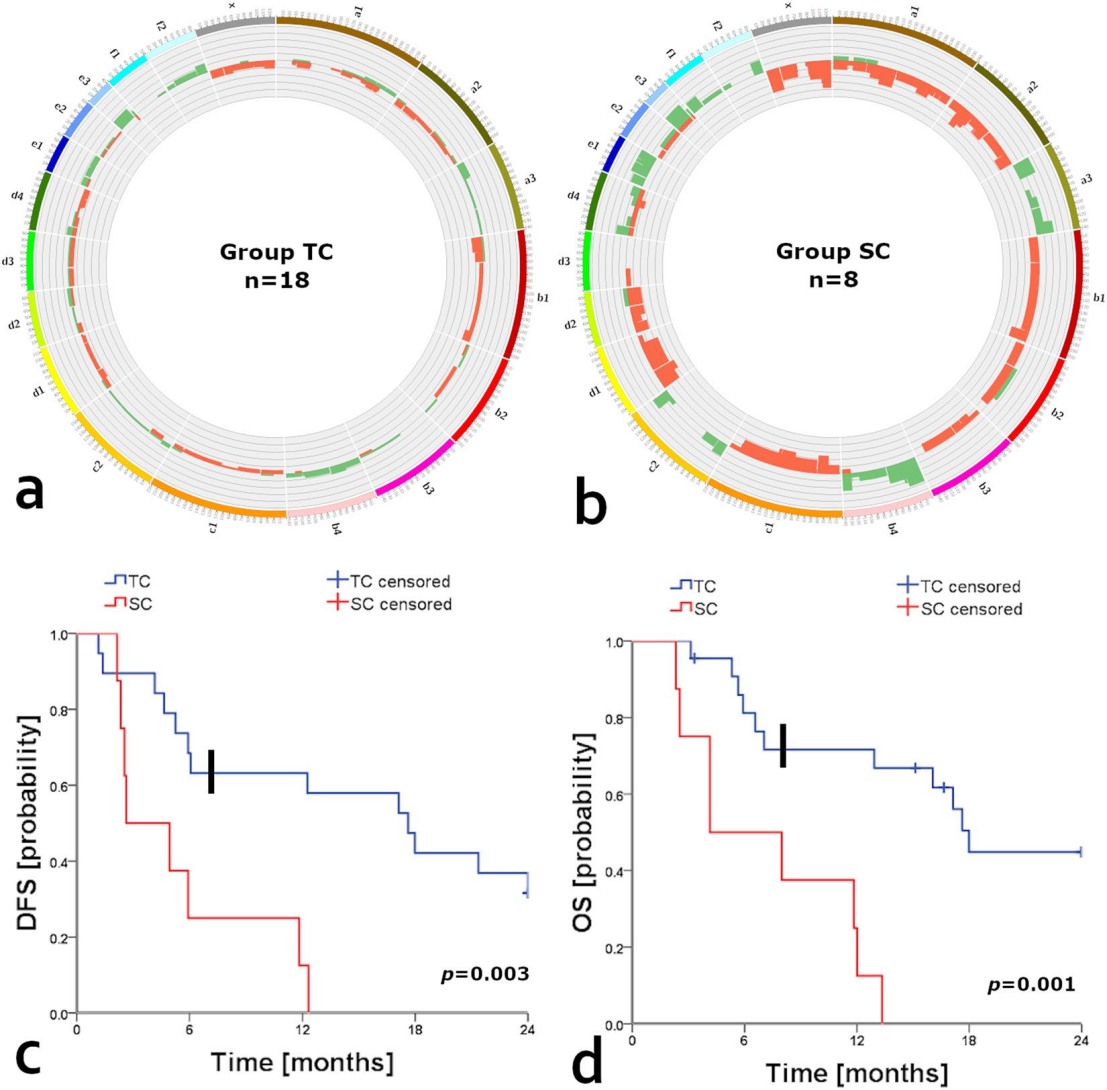
Comparative survival curves and circos plots. Comparative circos plots of (**a**) group TC (n = 18) and (**b**) group SC (n = 8); outer multicolour ring illustrates chromosome location, green and orange regions in the central ring pinpoint CNGs and CNLs, respectively. Kaplan-Meier curves of (**c**) DFS and (**d**) cancer-specific OS according to histological group (TC: tubulopapillary carcinomas, and SC: solid carcinomas and comedocarcinomas), in both plots events before the black bold vertical lines represent a subset of patients in the group TC displaying shorter survival intervals (DFS; *p* < 0.00001, and OS; *p* = 0.0004) in comparison to the rest of the cats allocated within the group TC. Kaplan-Meier curves adapted from Granados-Soler *et al*. https://d-nb.info/1191752739/34.

The DFS and cancer-specific OS of cats in the group SC were significantly lower (*p* = 0.003 and *p* = 0.001; respectively) than those of cats allocated in the group TC (Table 1). The lower frequency of CNVs in the group TC (Fig. 2a) compared with the group SC (Fig. 2b) corresponded with the higher survival rates observed in the group TC (Fig. 2c,d). However, a subset of patients (n = 7) in the group TC displayed shorter survival intervals (DFS; *p* < 0.00001, and OS; *p* = 0.0004) in comparison to the rest of the cats allocated in the group (Fig. 2c,d). Moreover, those patients mainly represented molecular subtypes with worst prognosis (e.g. LB *f*HER2−, and triple-negative) and were characterised by high-CNVs scores, high Ki-67 indexes, and a significantly (*p* = 0.001) higher percentage of overlapped aberrant genomic windows (mean [SD]: 27.02 [14.73] %). On the other hand, group TC included all LA subtype tumours characterised by lower histological malignancy grades (EE system), low Ki-67 indexes and higher survival intervals. In contrast, all patients included in the group SC displayed reduced survival intervals. Moreover, this group predominantly included molecular subtypes with worst prognosis (i.e. *f*HER2+, basal-like TN, and normal-like TN), high Ki-67 indexes, and high histological malignancy grades when applying the EE system.

**Table 1:** Specific survival rates according to histological group, molecular subtype, and CNVs score.

Variable	DFS months mean ± sem (n^*^)	*p*-value^**^	Cancer-specific OS months mean ± sem (n^*^)	*p*-value^**^
***Histological group***				
group TC	14.9 ± 2.1 (n = 13)	0.003	16.7 ± 1.7 (n = 11)	0.001
group SC	5.6 ± 1.5 (n = 8)		7.2 ± 1.6 (n = 8)	
***Molecular subtype***				
LA	21.2 ± 2.7 (n = 3)	<0.0001	23.1 (n = 1)	<0.00001
LB *f*HER2+	17.1 (n = 1)		17.1 (n = 1)	
LB *f*HER2−	12 ± 5.9 (n = 2)		14.3 ± 4.2 (n = 2)	
*f*HER2+	12.3 (n = 1)		13.3 (n = 1)	
normal-like TN	7.7 ± 2 (n = 8)		9.4 ± 1.7 (n = 8)	
basal-like TN	2.8 ± 0.6 (n = 5)		3.9 ± 0.7 (n = 5)	
***CNVs score***				
low-CNVs	19.6 ± 2 (n = 6)	0.0002	20.9 ± 1.2 (n = 4)	0.0002
high-CNVs	5.2 ± 1.1 (n = 12)		8.4 ± 1.2 (n = 12)	
low-CNGs	18.2 ± 2.4 (n = 7)	0.0002	19.6 ± 1.7 (n = 5)	0.01
high-CNGs	5.6 ± 1.1 (n = 11)		8.6 ± 1.3 (n = 11)	
low-CNLs	16.5 ± 2.1 (n = 9)	NS	18.4 ± 1.4 (n = 7)	0.05
high-CNLs	7.4 ± 2.4 (n = 9)		10.9 ± 2.4 (n = 9)	

^*^Group size after survival analysis censoring.

^**^Log Rank univariate (Mantel-Cox). TC, tubulopapillary carcinomas; SC, solid carcinomas and comedocarcinomas; LA, luminal A; LB *f*HER2−, luminal B *f*HER2 negative; LB *f*HER2+, luminal B *f*HER2 positive, *f*HER2+, *f*HER2 positive; and TN, triple-negative.

To explore survival differences observed within the patients allocated in group TC, we independently evaluated the survival intervals of patients distributed according to molecular subtype, amount of CNVs, and additional clinical and histopathological variables evaluated. Regardless of the histopathological diagnosis, patients showed important differences in survival times according to molecular subtype and CNVs score (Table 1).

### Univariate and multivariate analysis

In the univariate analysis, 17 CNVs (CNGs = 10, and CNLs = 7) among the 27 common CNVs (>20% of the cases) were significantly correlated with poor DFS, among them 10 CNVs (CNGs = 4, and CNLs = 6) were also associated with reduced cancer-specific OS, Supplementary Table 7. Moreover, high Ki-67 index (*p* = 0.0001), and lymph node metastasis at diagnosis (*p* = 0.001) were significantly associated with reduced DFS. On the other hand, cancer-specific OS was negatively influenced by high Ki-67 index (*p* < 0.0001), lymph node metastasis at diagnosis (*p* = 0.001), tumour size^8^ (*p* = 0.04), and clinical stage^8^ (*p* = 0.01).

Patients showed significant differences in survival intervals when applying the EE grading (DFS; *p* = 0.05 and cancer-specific OS; *p* = 0.02) and the MMEE grading (cancer-specific OS; *p* = 0.02). Survival intervals were significantly different between patients with well-differentiated (grade I) and poorly-differentiated (grade III) tumours using the EE (DFS, *p* = 0.04; and cancer-specific OS, *p* = 0.005) and the MMEE (cancer-specific OS, *p* = 0.001) grading systems. However, survival intervals between grades I and II, and grades II and III were not significantly different when applying any of the histological grading systems employed (EE, MMEE, and Mills-2015). All additional independent variables considered including breed, age, and reproductive status did not influence DFS and cancer-specific OS (*p* > 0.05).

In the multivariate analysis, CNGs in F2 64–82.3 Mb (*p* = 0.004), CNGs in B4 1–29 Mb (*p*=0.023), and CNLs in FCA B1 1–23 Mb (*p* = 0.002) remained significantly associated with reduced DFS. On the other hand, CNGs F2 64–82.3 Mb (*p* = 0.008), CNGs B4 1–29 Mb (*p* = 0.024), and high-Ki-67 index (*p* = 0.02) remained significantly associated with poor cancer-specific OS, Table 2.

**Table 2:** Significant variables in univariate and multivariate analysis.

DFS univariate		DFS multivariate		Cancer-specific OS univariate		Cancer-specific OS multivariate	
DFS (months)	*p*-value	HR (95% CI)	*p*-value	OS (months)	*p*-value	HR (95% CI)	*p-*value
***CNGs F2 64–82***.***3 Mb***							
absent 16.1 ± 2.1 (n = 10)	0.0002	1.7 (0.3–7.8)	0.004	absent 18.3 ± 1.5 (n = 8)	<0.00001	18.2 (2.1–157)	0.008
present 3.8 ± 0.7 (n = 8)				present 5.2 ± 0.7 (n = 8)			
***CNGs FCA B4 1–29 Mb***							
absent 16.1 ± 2.3 (n = 9)	0.001	8.4 (1.3–53.2)	0.02	absent 17.4 ± 1.9 (n = 7)	0.01	25.6 (1.5–424.8)	0.02
present 5.1 ± 1.4 (n = 9)				present 8.9 ± 1.5 (n = 9)			
***CNLs B1 1–23 Mb***							
absent 15.4 ± 2.1 (n = 11)	<0.0001	23.5 (3.1–174.9)	0.002	absent 17.4 ± 1.6 (n = 9)	0.0003	—	NS
present 3.4 ± 0.7 (n = 7)				present 6.1 ± 1.3 (n = 7)			
***Ki-67 index***^34^							
low Ki-67 21.1 ± 2.1 (n = 2)	0.0001	—	NS	low Ki-67 21.5 ± 2.4 (n = 3)	<0.0001	15.1 (1.5–143.2)	0.02
high Ki-67 9.6 ± 1.2 (n = 18)				high Ki-67 7.4 ± 1.3 (n = 18)			

HR; hazard ratio, CI; confidence interval, and NS; not significant.

### Analysis of common CNGs significantly correlated with reduced survival intervals

CNGs influencing poor outcome encompassed genes frequently amplified in HBCs including transcription factors *GATA3*, *GATA5*, *MLLT10*, and *TAF3*, cell cycle regulators (e.g. *CCND2*), proto-oncogenes (e.g. *SRC*), and epithelial to mesenchymal transition (EMT) markers (e.g. *VIM*, *LAMA5*, and *ZEB1*)^15,19,21,28,41–43^, Table 3. Genes in CNGs enriched the endocytosis pathway (Table 4), including cancer-related genes *STAM*, *RAB22A*, *PIP5K1B*, *ASAP1*, and *ARFGAP1* involved in early endosomes formation, membrane trafficking regulation, and internalising and recycling of junctional proteins (e.g. cadherins)^44–49^. In addition, CNGs comprised multiple tight junction components including cell-polarity regulators (e.g. *PARD6B*) and tyrosine kinases (e.g. *SRC*, *PRKCQ*, and *CSNK2A1*) with major roles in the disruption of adherent junctions, anoikis resistance, and cell migration^45,49,50^ (Table 4).

**Table 3:** Common CNGs associated with reduced DFS and cancer-specific OS.

CNG (Mb)	*p-*value^‡^		Relevant genes
	DFS	OS	
A3 1–31 (n = 8)	0.003	0.002	*TPX2*, *DLGAP4*, *DSN1*, *RBL1*, *FAM83D*, *MYBL2*, *PABPC1L*, *UBE2C*, *CTSA*, *MMP9*, *SULF2*, *CSE1L*, *SALL4*, *ZNF217*, *PFDN4*, *AURKA*, *RAE1*, *PMEPA1*, MYT1^*^, SLC2A4RG^*^, ZGPAT^*^, PTK6^*^, EEF1A2^*^, KCNQ2^*^, CHRNA4^*^, ARFGAP1^*^, *GATA5*, RPS21^*^, LAMA5^*,***^, GNAS^*^, TMEPAI^*^, ZBP1^*^, PCK1^*^, TFAP2C^*^, CSTF1^*^, DOK5^*^, CYP24A1^*^, BCAS1^*^, ZNF217^*^, SRC^*^
B4 1–29^†^ (n = 9)	0.001	0.01	KIN^*^, TAF3^*^, GATA3^*^, *MCM10*, VIM^***^, *MLLT10*, ZEB1^*,***^
D4 1–16.7 (n = 6)	0.009	0.006	*OMD*, *SYK*, *PDCD1LG2* (*PD-L2*), *CD274* (*PD-L1*), *JAK2*, *GLIS3*, *RFX3*, *DMRT1*, *DOCK8*, *FOXD4*
F2 64–82.3^†^ (n = 8)	0.0002	0.006	*EXT1*, *SAMD12*, *NOV*, *TAF2*, *DCC1*, *MRPL13*, *MTBP*, *SNTB1*, *HAS2*, *ZHX2*, *FBXO32*, *RNF139*, *TATDN1*, *MTSS1*, MYC^*,**^, *WISP1*, *NDRG1*, *ST3GAL1*, *KHDRBS3*, *PTK2*

DFS, disease-free survival; OS, cancer-specific overall survival.

^*^Breast cancer-associated CNGs^19,28,41–43^.

^**^Amplification validated by the cancer gene census in human cancers^59^.

^***^EMT-related genes commonly affected by CNGs in multiple cancer types^21^.

^†^significant in multivariate analysis, and ^‡^univariate analysis log-rank (Mantel-Cox).

**Table 4:** Functional clustering and KEGG pathway analysis of genes in CNGs significantly correlated with poor outcome.

Biological process	Genes	*p*-value
***KEGG pathway analysis***		
Endocytosis	*ARFGAP1*, *PARD6B*, *IL2RA*, *CHMP4B*, *RAB22A*, *PIP5K1B*, *ASAP1*, *STAM*, *ITCH*, *SRC*	0.003
Tight junction	*PRKCQ*, *PARD6B*, *CSNK2A1*, *EPB41L1*, *TJP2*, *MYH7B*, *SRC*, *MYL9*	0.004
Cell adhesion molecules (CAMs)	*SIGLEC1*, *ITGA8*, *CD274* (*PD-L1*), *CD40*, *SDC4*, *CDH4*, *PDCD1LG2* (*PD-L2*)	0.004
Pathways in cancer	*E2F1*, *PLCG1*, *LAMA5*, *MMP9*, *BCL2L1*, *STK4*, *MYC*	0.04
***Functional clustering***		
WAP	*WFDC8*, *PI3*, *SLPI*, *WFDC5*, *WFDC2*, *WFDC3*, *WFDC13*	<0.00001
Serine protease inhibitor^†^	*WFDC8*, *SPINT4*, *PI3*, *ITIH5*, *SLPI*, *ITIH2*, *WFDC5*, *WFDC2*, *WFDC3*	0.001
Protease inhibitor^‡^	*WFDC8*, *SPINT4*, *PI3*, *SERPINE1*, *ITIH5*, *SLPI*, *ITIH2*, *R3HDML*, *WFDC5*, *WFDC2*, *WFDC3*	0.02
Serine-type endopeptidase inhibitor activity^‡^	*WFDC8*, *SPINT4*, *PI3*, *ITIH5*, *SLPI*, *ITIH2*, *WFDC5*, *WFDC2*, *WFDC3*	0.007
Enzyme inhibitor activity^‡^	*WFDC10B*, *PHACTR3*, *PKIG*, *ANXA1*, *TRIB3*, *BCL2L1*, *R3HDML*, *TRIB1*, *WFDC13*, *WFDC8*, *SPINT4*, *PI3*, *SLPI*, *ITIH5*, *ITIH2*, *WFDC5*, *WFDC2*, *WFDC3*	0.004

^†^GOTERM.

^‡^SP_PIR_KEYWORDS, and KEGG; Kyoto Encyclopaedia of Genes and Genomes. Data analysed with DAVID Bioinformatics.

Among amplified regions, A3 1–31 Mb resemble some of the most common CNGs reported in multiple human cancers (HSA 20q)^51,52^. A3 1–31 Mb included all genes comprised in a gene signature of 18 genes (i.e. *TPX2*, *DLGAP4*, *DSN1*, *RBL1*, *FAM83D*, *MYBL2*, *PABPC1L*, *UBE2C*, *CTSA*, *MMP9*, *SULF2*, *CSE1L*, *SALL4*, *ZNF217*, *PFDN4*, *AURKA*, *RAE1*, and *PMEPA1*) associated with poor prognosis in many human cancer types^51^. Moreover, this region harboured genes encoding proteins with a whey-acidic-protein (WAP) motif including putative human cancer biomarkers *SLPI*, *PI3*, and *WFDC2*^51,53^, and several Cell Adhesion Molecules (CAMs), for instance, metastasis promoters *CDH4* and *SDC4*^49,54,55^, Table 4. Several genes in A3 1–31 Mb significantly enriched pathways in cancer (Table 4) including the transcription factor *E2F1* recently identified as a key regulator of a set of metastasis promoter genes in HBCs^56^. Moreover, A3 1–31 Mb, and B4 1–29 Mb (significant in multivariate analysis) harboured multiple genes (e.g. *MYLK2*, *MYL9*, *ITGA8*, and *PIP4K2A)* enhancing cellular movement and migration-related pathways, Table 4. An additional CNG located in FCA D4 1–16.7 Mb (human homologue region located in HSA 9p) comprised genes mediating a broad variety of immune and inflammatory responses such as *CD274* (*PD-L1*) and *PDCD1LG2* (*PD-L2*) which co-gain characterises a subset of malignant solid tumours (including triple-negative HBCs) susceptible to immunotherapy through the inhibition of the *PD-L1/PD-1* immune checkpoint^57,58^.

F2 64–82.3 Mb (human homologue region located in HSA 8q) was significant in multivariate analysis and harboured several breast cancer-related genes affected by CNGs in HBCs^19,28,41^ including EMT-related genes (i.e. *MYC*, *SCRIB*, *NDRG1*, and *PTK2*) commonly affected by CNGs in multiple human cancer types^21^ (Tables 3 and 4), among them, *MYC* amplification is validated as somatic-CNG by the Cancer Gene Census (CGC)^59^. Moreover, this genomic region harboured *ASAP1* which encodes an oncoprotein correlated with enhancing cell motility, invasiveness, and poor survival in human cancers including HBCs^60–63^.

Besides CNGs influencing both DFS and cancer-specific OS, FCA E3 1–34.5 Mb (human homologue region located in HSA 7p) was only correlated with poor DFS and was the most common CNG detected (Supplementary Table 6). This CNG harboured multiple members of the leukocyte transendothelial migration pathway, including cell junction components (e.g. *CLDN3*, *CLDN4*, and *CLDN15*), and serine- and threonine-specific protein kinases (e.g. *PRKCB*). Moreover, CNGs in FCA E3 1–34.5 Mb included genes (e.g. *MYL10*, *ACTB*, *RAC1*, and *MYLPF*) involved in cell movement, actin cytoskeleton remodelling, and cellular proliferation and motion such as *SBDS*, *ITGAM*, *PDGFA*, and *RAC1*. Among them, *RAC1* actively promotes cell motility in breast cancer cells^64,65^ and *PDGFA* has been reported as highly expressed in FMCs and derived cell lines^66^. Furthermore, E3 1.1–34.5 Mb harboured *SERPINE1*; a gene encoding a cell adhesion protein involved in invasion and metastasis promotion in different human malignancies including HBCs^67–69^

### Analysis of common CNLs significantly correlated with reduced survival intervals

Common CNLs negatively influencing DFS and cancer-specific OS harboured the feline orthologous of multiple HBC-related genes reported as commonly deleted including *FOXP1*, *FBXO25*, *CSMD1*, *AGPAT5*, *PCM1*, *PTPRG*, and *PDGFRL*^15,19,27,28,41–43^ (Table 5). Genes in CNLs enriched lipid metabolism processes including PPAR-alpha targets such as *APOA1*, *APOA5*, *APOC3*, and *MMP1*^49^, Table 6. Moreover, deleted regions included several key genes (i.e. *SUCLG2*, *SDHD*, *DLAT*, and *PDHB*) participating in mitochondrial functions and citrate cycle regulation^49^. On the other hand, additional deleted genes (i.e. *TUSC3*, *STT3A*, *DPAGT1*, and *ALG9*) characterising the abnormal re-programming of metabolic pathways observed, enriched the N-Glycan biosynthesis pathway, Table 6.

**Table 5:** Common CNLs associated with reduced DFS and cancer-specific OS.

CNL (Mb)	*p* value^‡^		Relevant genes
	DFS	OS	
A2 23–37.3 (n = 8)	0.009	0.01	*WNT5A*, *FHIT*, PTPRG^*^, C3orf14^*^, *MAGI1*, *MITF*, *ACOX2*, *SUCLG2*, *PDHB*
A2 37.3–48 (n = 7)	0.04	0.05	FOXP1^*^, *RYBP*, *PPP4R2*, *PDZRN3*, *CHL1*, CNTN6^*^,*CNTN3*, *CNTN4*
B1 1–23^†^ (n = 7)	<0.00001	<0.0001	FBXO25^*^, CSMD1^*^, *ANGPT2*, AGPAT5^*^, *VEGFC*, *NEIL3*, *CASP3*, SORBS2^*^,*FAT1*, ASAH1^*^, *IRF2*, PCM1^*^, MTUS1^*^, PDGFRL^*^, SLC7A2^*^, MTMR7^*^, VPS37A^*^, CNOT7^*^, ZDHHC2^*^, MSR1^*^,TUSC3^*^, *ACSL1*, *RHOBTB2* (*DBC2*)^*^
D1 1.1–20 (n = 6)	0.006	0.018	*MMP7*, *PDGFD*, *CASP4*, ATM^*,**^, *SIK2*, *CRYAB*, *CLDN25*, *DDX10*, *POU2AF1*, *SDHD*, *ZBTB16*, *PAFAH1B2*, *PCSK7*, *KMT2A*, *DDX6*, *CBL*, *ARHGEF12*, *TECTA*, *MPZL3*, *MPZL2*, *CADM1*, *DSCAML1*, *MCAM*, *THY1*, *NCAM1*, *APOA4*, *APOA1*, *APOA5*, *APOC3*, *MMP1*, *DLAT*, *DPAGT1*, *ALG9*
D1 21–39.4 (n = 8)	<0.0001	0.0004	*ROBO3*, *ROBO4*, *HEPACAM*, *STT3A*, *CHEK1*, *ETS1*, *FLI1*, *NFRKB*, *ST14*, *ADAMTS15*, *OPCML*, *PGR*, *MAML2*, *OPCML*, *CNTN5*, *ESAM*, *NTM*, *BARX2*, *CDON*, *FEZ1*
D1 41.2–97 (n = 6)	0.001	0.004	*CD44*, *FZD4*, *PRSS23*, *ME3*, *EED*, *PICALM*, *SYTL2*, *CREBZF*, *ANKRD42*, *PCF11*, *RAB30*, *PRCP*, *TENM4*, *NARS2*, *GAB2*, *USP35*, *KCTD21*, *INTS4*, *AAMDC*, *RSF1*, *CLNS1A*, *AQP11*, *PAK1*, *GDPD4*, *OMP*, *CAPN5*, *WNT11*, *UVRAG*, *DGAT2*, *MAP6*, *SERPINH1*, *GDPD5*, *KLHL35*, *RPS3*, *ARRB1*, *NEU3*, *POLD3*, *PPME1*, *UCP2*, *RAB6A*, *RELT*, *FCHSD2*, *ATG16L2*, *CLPB*, *PHOX2A*, *INPPL1*, *FOLR1*, *NUMA1*, *NUP98*, *LMO1*, *NAV2*, *DBX1*, *SLC6A5*, *NELL1*, *CAPRIN1*, *NAT10*, *ABTB2*, *CAT*, *ELF5*, *EHF*, *HRAS*, *FANCF*, WT1^**^, *LMO2*

FCA, feline chromosome; DFS, disease-free survival; cancer-specific OS, overall survival; TC, tubulopapillary carcinoma group; SC, solid carcinoma and comedocarcinoma group; and NS, non-significant.

^*^Breast cancer-associated CNLs^19,27,28,41–43^.

^**^Deletion validated by the cancer gene census in human cancers^59^.

^†^Significant variable in multivariate analysis, and ^‡^Log Rank (Mantel-Cox).

**Table 6:** Genes in genomic regions affected by common CNLs that significantly cluster for specific biological processes and KEGG pathways.

Biological process	Genes	*p*-value
***KEGG pathway analysis***		
Pyruvate metabolism	*LDHC*, *LDHA*, *LDHAL6A*, *DLAT*, *ACAT1*, *PDHB*	0.002
PPAR signalling pathway	*ACOX2*, *APOA1*, *ACSL1*, *ILK*, *APOA5*, *APOC3*, *MMP1*	0.006
N-Glycan biosynthesis	*TUSC3*, *STT3A*, *DPAGT1*, *ALG8*, *ALG9*	0.006
***Functional clustering***		
Cell adhesion^†^	*MPZL3*, *MPZL2*, *CADM1*, *OPCML*, *CNTN5*, *INPPL1*, *CNTN6*, *DSCAML1*, *MCAM*, *DCHS1*, *NCAM1*, *CD44*, *FAT3*, *HEPACAM*, *FAT1*, *ESAM*, *CNTN4*, *CNTN3*, *CHL1*, *NTM*	0.004
Biological adhesion^†^	*MPZL3*, *MPZL2*, *CADM1*, *OPCML*, *DSCAML1*, *DCHS1*, *APOA4*, *BARX2*, *FAT3*, *CD44*, *FAT1*, *ILK*, *ESAM*, *TECTA*, *CNTN5*, *MAGI1*, *INPPL1*, *CNTN6*, *MCAM*, *THY1*, *NCAM1*, *HEPACAM*, *CDON*, *CNTN4*, *CNTN3*, *CHL1*, *NTM*, *FEZ1*	0.01
Proteinaceous extracellular matrix^†^	*TECTA*, *WNT5A*, *ADAMTS15*, *MMP8*, *MMP27*, *MMP7*, *MMP13*, *MMP1*, *MMP12*, *MMP10*, *ADAMTS9*, *MMP20*, *ADAMTS8*, *CHL1*	0.05
Cell-cell adhesion^†^	*MPZL2*, *CADM1*, *DSCAML1*, *DCHS1*, *THY1*, *APOA4*, *NCAM1*, *BARX2*, *FAT3*, *CD44*, *FAT1*, *CNTN4*, *ESAM*	0.05

^†^GOTERM. Data analysed with DAVID Bioinformatics.

CNLs included a considerable amount of genes involved in specific biological processes (i.e. cell adhesion, biological adhesion, and cell-cell adhesion) that would normally prevent cellular migration, Table 6. Among deleted regions correlated with poor outcome, CNLs affecting the proximal region of FCA A2 (23–37.3 Mb, and 37.3–48 Mb; human homologue regions located in HSA 3p) comprised genes implicated in cell and biological adhesion, including several contactins (e.g. *CNTN3*, *CNTN4*, and *CNTN4*) active during embryonic development^49^, Tables 5 and 6. On the other hand, CNLs in FCA B1 1–23 Mb (human homologue region located in HSA 8p) were significant in multivariate analysis and harboured multiple tumour suppressors (e.g. *CSMD1*, *MSR1*, *MTUS1*, and *TUSC3*) previously correlated with poor prognosis in HBCs^15,19,23,27,70^. Deleted genes predominantly detected in proximal FCA D1 (1–20 Mb, 21–39.4 Mb, and 41.2–97 Mb; human homologue regions located in HSA 11q) encoded extracellular matrix proteins and membrane-associated guanylate kinases (e.g. *TECTA*, and *MAGI1*), and cancer-associated CAMs (e.g. *CADM1*, *OPCML*, *MCAM*, *HEPACAM*, and *ESAM*)^49^, Table 6. Moreover, deleted regions affecting FCA D1 included genes validated as cancer variants by the CGC^59^ including *ATM* validated as somatic-CNL, and tumour suppressor *WT1* validated somatic- and germline-CNL.

### CNVs distribution across molecular subtypes

**Figure 3:**
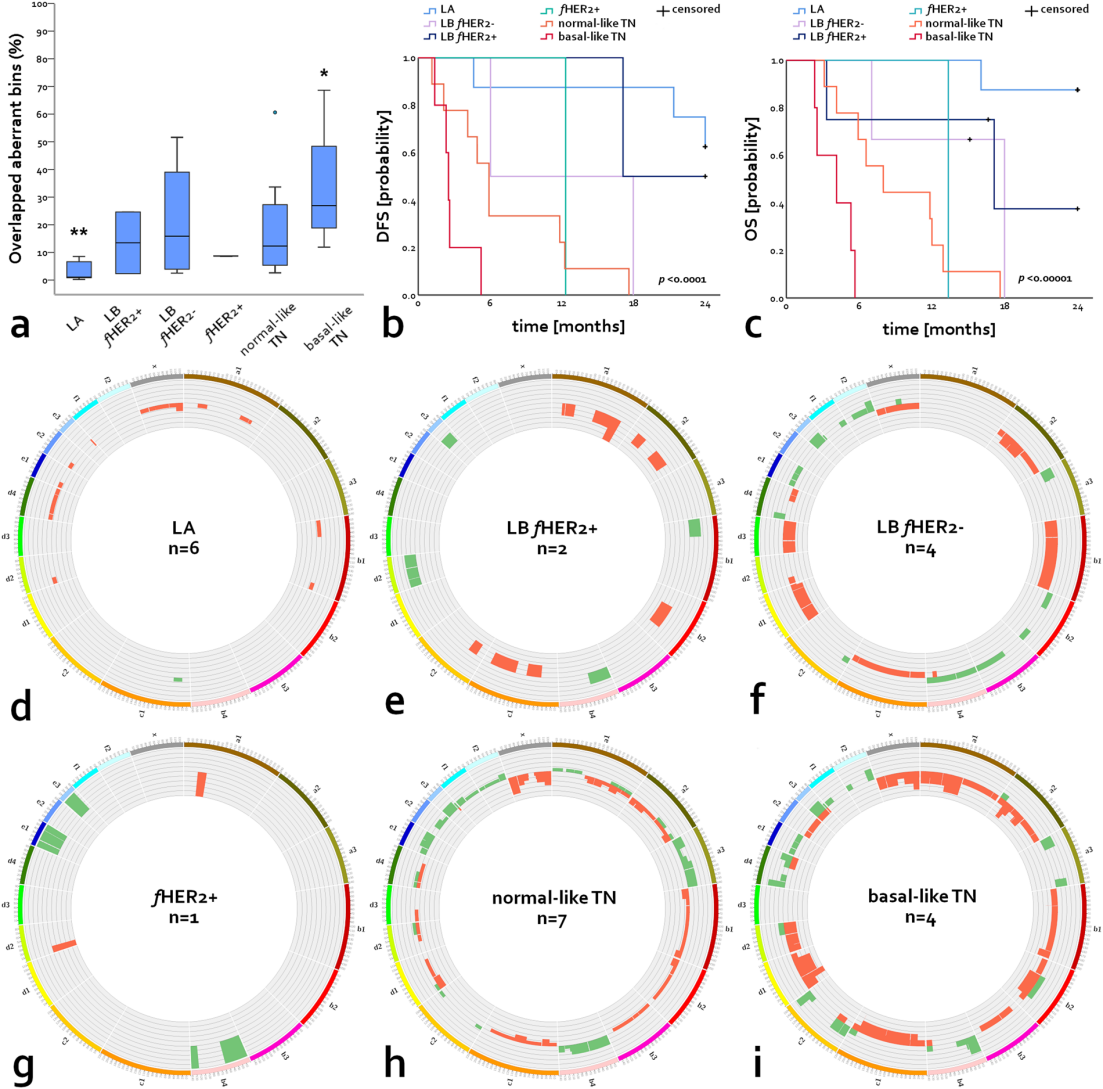
CNVs landscape and associated survival of FMCs according to molecular subtype. (**a**) Percentage of overlapped aberrant genomic windows according to molecular subtype; data are displayed as mean (SD); *p < 0.05, **p < 0.01, and ***p < 0.001. (**b**) DFS and (**c**) cancer-specific OS Kaplan-Meier curves according to molecular subtype. Comparative circos plots of (**d**) LA subtype, (**e**) LB *f*HER2+, (**f**) LB *f*HER2−, (**g**) *f*HER2+, (**h**) normal-like TN, and (**i**) basal-like TN. Outer multicolour ring illustrates chromosome location, green and orange regions in the central ring pinpoint CNGs and CNLs, respectively. LA, luminal A; LB *f*HER2+, luminal B *f*HER2-positive; LB *f*HER2−, luminal B *f*HER2-negative; *f*HER2+, *f*HER2-positive; and TN, triple-negative.

We observed a negative correlation between the percentage of aberrant genomic windows (Fig. 3a) and survival intervals (Fig. 3b,c). Among all subtypes studied, LA subtype tumours displayed the highest survival intervals (Fig. 3b,c), and the significantly (*p* = 0.001) lowest percentage of aberrant genomic windows affected by CNVs (n = 6, mean [SD]: 3.05 [3.6] %, Fig. 3a). This subtype exhibited a very low occurrence of CNVs (Fig. 3d) in comparison with the other molecular subtypes. Moreover, all patients within the group had a low-CNVs score, Supplementary Table 5. Only CNLs in FCA X (108–126.3 Mb) affected two patients simultaneously (Fig. 3d), all additional aberrations affected different patients individually. Aberrations detected in LA subtype tumours, included one CNG in proximal FCA C1 (21–45 Mb) and multiple CNLs distributed unevenly along FCAs A1, B1, D2, E1, E2, E3, and whole chromosomes D4 and X (Fig. 3d). CNVs affecting LA subtype tumours included two out of the ten CNVs associated with poor survival intervals (CNLs in B1 1–23 Mb and CNGs in D4 1–16.7 Mb), however, both of them were detected in only one case. Interestingly, none of the patients within the group harboured the most common CNV observed in this study (CNGs in FCA E3 1–34.5 Mb).

The two cases classified as LB *f*HER2+ (Fig. 3e) displayed a different pattern of aberration, one case was classified as low-CNVs (Supplementary Table 5) score and carried CNLs affecting FCAs A1 and A2. In contrast, the other case was classified as high-CNVs (Supplementary Table 5) and carried CNLs in FCAs A1, A2, B2, C1 and C2, and CNGs affecting FCAs B1, B4, D2, and E3. Interestingly, this case was the only one among all cases included in this study carrying CNGs affecting B1 (1–23 Mb). Only CNLs in FCA A1 (179–194 Mb and 198–203.7 Mb) affected both LB *f*HER2+ cases (Fig. 3e), however, none of them was correlated with reduced survival intervals.

Among LB *f*HER2− cases (n = 4), two cats displayed low-CNVs scores and two high-CNVs scores, Supplementary Table 5. CNVs affected all FCAs except A1 and E2, nevertheless, most of the structural rearrangements affected patients individually and none of them affected more than two patients simultaneously (Fig. 3e). Aberrations affecting two patients within this subtype included the ten CNVs correlated with reduced survival intervals listed in Tables 3 and 5.

The only case classified as *f*HER2+ exhibited nine CNGs affecting FCAs B4, E1, and E3, and two CNLs in FCAs A1 and D2 (Fig. 3g). Eight out of the nine CNGs were correlated with reduced DFS or cancer-specific OS and four of them (B4 1–29 Mb and E1 [19.3–37 Mb, 38–44 Mb, and 44–62 Mb]) affected both. On the other hand, none of the CNLs detected was correlated with poor outcome.

Normal-like TN-FMCs (n = 7) included three cases scored as low- and four as high-CNVs, Supplementary Table 5. Despite the poor survival observed and the high amount of CNVs detected (n = 77) in this subtype, almost half of them (n = 32) affected patients individually (Fig. 3h). Nineteen CNVs affected more than 40% (3/7) of the patients. Among them, ten negatively influenced DFS, and three (CNGs in FCAs A3 1–31 Mb, and B4 1–29 Mb; and CNLs in FCA D1 1.1–20 Mb) affected both DFS and cancer-specific OS. In this subtype, CNLs in FCA X (1–26 Mb and 108–126.3 Mb) were the most common (five and four patients; respectively). However, none of them was correlated with reduced survival intervals.

Finally, all basal-like TN-FMCs (n = 4) were classified as high-CNVs score, Supplementary Table 5. Moreover, this subtype exhibited the significantly (*p* = 0.05) highest amount of aberrant genomic windows (Fig. 3a) and the lowest survival intervals among all molecular subtypes studied (Fig. 3b,c). Basal-like TN-FMCs displayed 78 structural rearrangements affecting all chromosomes but D3 (Fig. 3i). CNVs affecting this subtype included all rearrangements correlated with reduced survival intervals listed in Tables 3 and 5, and Supplementary Table 7. Among common structural rearrangements correlated with reduced DFS and cancer-specific OS, CNGs in FCAs B4 (1–29 Mb) and D4 (1–16.7 Mb) and CNLs in A2 (23–37.3 Mb), and D1 (21–39.4 Mb, and 41.2–97 Mb) were detected in 75% (3/4) of the cases. Among them, CNLs affecting FCA D1 (21–39.3 Mb) affected all patients included in this group (Fig. 3i) and was correlated with reduced DFS and cancer-specific OS (Table 5). Apart from common CNVs (>20% of the cases) correlated with poor outcome in the whole population, two additional CNLs affected three out of the four basal-like TN cases and negatively influenced survival intervals as follows: A1 105–124 Mb (DFS, *p* = 0.002, and OS *p* < 0.0001; normal-like TN = 2 and basal-like TN = 3), and D2 54–88.9 Mb (DFS, *p* = 0.009, and OS *p* = 0.001; LB *f*HER2+ = 1, normal-like TN = 2, and basal-like TN = 3).

### Functional clustering analysis of genes in CNVs characterising basal-like TN-FMCs

Considering the poor survival characterising basal-like TN-FMCs and the high frequency of CNVs affecting this subtype, common CNVs within the group (>75% of the cases) influencing both DFS and OS were independently analysed. Accordingly, genes in CNGs (B4 [1–29 Mb] and D4 [1–16.7 Mb]) and CNLs (A1 [105–124 Mb], A2 [23–37.3 Mb and 37.3–48 Mb], D1 [21–39.3 Mb and 41.2–97 Mb] and D2 [54–88.9 Mb] were assessed using functional clustering and KEGG pathway analyses to detect cancer-related genes and identify deregulated biological processes characterising the poor prognosis observed in this subtype.

After KEGG pathway analysis, genes (i.e. *IL2RA*, *IL15RA*, *JAK2*, and *STAM*) in CNGs enriched the Jak-STAT signalling pathway, Table 7. Moreover, CNGs affecting FCA B4 1–29 Mb (significant in multivariate analysis) encompassed important cancer-associated kinases (i.e. *MAP3K8*, *PFKFB3*, and *PRKCQ*), and several genes encoding kinases, transferases, and protein serine/threonine kinases involved in cellular electrolyte homeostasis and metabolic reprogramming (i.e. *WNK2*, and *PFKP*), mitosis regulation (i.e. *MASTL*, and *PFKFB3*), and actin cytoskeletal functions (i.e. *MYO3A*)^49^. Similarly, CNGs in FCA D4 (1–16.7 Mb) harboured protein serine/threonine kinase-coding genes implicated in the development of multiple cancer types in humans (i.e. *CKS2*, *JAK2*, and *SYK*)^49^. Both amplified regions included several genes coding for proteins with sh3 domain (i.e. *CACNB2*, *ABI1*, *MPP7*, *STAM*, *TJP2*, and *NEBL*). Among them, *ABI1*, *MPP7*, and *NEBL* participate in different processes related to cellular communication and motility (e.g. membrane trafficking, actin cytoskeleton remodelling, and epithelial cell polarity) through interactions with different tyrosine kinases^49^, Table 7.

**Table 7:** Functional clustering and KEGG pathway analysis of genes in common CNGs detected in basal-like TN-FMCs.

Biological process	Genes	*p*-value
KEGG pathway analysis of genes in CNGs^*^		
Jak-STAT signalling pathway	*IL2RA*, *IL15RA*, *JAK2*, *STAM*	0.05
Functional clustering of genes in CNGs^*^		
Kinase^‡^	*TRPM6*, *SEPHS1*, *MYO3A*, *PFKFB3*, *AK3*, *PFKP*, *PIP5K1B*, *WNK2*, *PRKCQ*, *MAP3K8*, *CKS2*, *ROR2*, *JAK2*, *MASTL*, *PIP4K2A*, *CDK20*, *SYK*, *CAMK1D*	<0.00001
Transferase^‡^	*TRPM6*, *SPTLC1*, *SEPHS1*, *MYO3A*, *PFKFB3*, *MTPAP*, *PIP5K1B*, *AK3*, *PFKP*, *WNK2*, *PDSS1*, *SUV39H2*, *NMT2*, *PRKCQ*, *NSUN6*, *ST8SIA6*, *PTAR1*, *TRDMT1*, *MAP3K8*, *ROR2*, *JAK2*, *MASTL*, *PIP4K2A*, *CDK20*, *CAMK1D*, *SYK*	<0.00001
Serine/threonine-protein kinase^†,‡^	*PRKCQ*, *TRPM6*, *MYO3A*, *MAP3K8*, *WNK2*, *MASTL*, *CDK20*, *CAMK1D*	0.04
Protein kinase, ATP binding site^†^	*PRKCQ*, *MYO3A*, *MAP3K8*, *ROR2*, *JAK2*, *WNK2*, *MASTL*, *CDK20*, *SYK*, *CAMK1D*	0.02
Protein kinase activity^†^	*PRKCQ*, *TRPM6*, *MYO3A*, *MAP3K8*, *ROR2*, *JAK2*, *WNK2*, *MASTL*, *CDK20*, *SYK*, *CAMK1D*	0.05
sh3 domain^§,‡,#^	*CACNB2*, *ABI1*, *MPP7*, *STAM*, *TJP2*, *NEBL*	0.01

^*^CNGs in FCAs B4 (1–29 Mb) and D4 (1–16.7 Mb).

^†^GOTERM.

^‡^SP_PIR_KEYWORDS.

^§^UP_SEQ_FEATURE.

^#^INTERPRO. Data analysed with DAVID Bioinformatics.

On the other hand, genes in CNLs enriched the Wnt receptor signalling pathway (functional clustering and KEGG pathway analyses) and the homeobox family, Table 8. Genes in CNLs significantly enriched the basal cell carcinoma pathway including several members of the Wnt signalling pathway (e.g. *WNT5A*, *TCF7*, *WNT11*, *WNT8A*, and *WNT8B*), Table 8. Furthermore, multiple enzyme-coding genes (e.g. *LDHC*, *LDHA*, *ALDH7A1*, and *LDHAL6A*) in CNLs enriched different metabolic pathways (e.g. pyruvate, and propanoate metabolism). Interestingly, genes in CNLs were involved in different biological processes normally preventing cellular movement and tumour dissemination, including several components of the proteinaceous extracellular matrix, protocadherins (e.g. *PCDH1*, *PCDHB8*, and *PCDHB1*), and cell adhesion molecules (e.g. *OPCML*, *CTNNA1*, *MEGF10*, *PCDH1*, *FAT3*, *HEPACAM*, *ESAM*, *NTM*, and *SPON1*)^49^, Table 8. Additionally, two basal-like TN-FMCs had CNLs in A1 1–28 Mb harbouring *BRCA2* reported as somatic- and germline-CNV by the CGC in HBCs^59^; interestingly, this aberration was not observed in any other subtype.

**Table 8:** Functional clustering and KEGG pathway analysis of genes in common CNLs detected in basal-like TN-FMCs.

Biological process	Genes	*p*-value
KEGG pathway analysis of genes CNLs^*^		
Wnt signalling pathway	*WNT5A*, *TCF7*, *CTBP2*, *BTRC*, *SKP1*, *FZD4*, *TCF7L2*, *SFRP5*, *PPP2CA*, *PRICKLE2*, *FRAT1*, *FRAT2*, *WNT11*, *WNT8A*, *WNT8B*	0.002
Basal cell carcinoma	*WNT5A*, *TCF7*, *WNT11*, *FZD4*, *TCF7L2*, *WNT8A*, *SUFU*, *WNT8B*	0.006
Pyruvate metabolism	*LDHC*, *LDHA*, *ALDH7A1*, *ME3*, *LDHAL6A*, *PDHB*	0.01
Propanoate metabolism	*LDHC*, *LDHA*, *ALDH7A1*, *SUCLG2*, *LDHAL6A*	0.03
Functional clustering of genes in CNLs^*^		
Wnt receptor signalling pathway^†,‡^	*WNT5A*, *TCF7*, *LZTS2*, *BTRC*, *LDB1*, *MITF*, *FZD4*, *UBE2B*, *TCF7L2*, *SFRP5*, *DKK3*, *HHEX*, *FBXW4*, *FRAT1*, *FRAT2*, *ZRANB1*, *WNT11*, *WNT8A*, *WNT8B*	<0.00001
Homeobox^‡,§^	*PHOX2A*, *HMX2*, *LBX1*, *EMX2*, *PAX6*, *VAX1*, *HESX1*, *PKNOX2*, *HHEX*, *BARX2*, *NKX1-2*, *POU4F3*, *PITX3*, *HMX3*, *PITX1*, *TLX1*, *NKX2-3*	0.003
Proteinaceous extracellular matrix^†,‡^	*WNT5A*, *ADAMTS19*, *CRTAC1*, *ADAMTS15*, *SPOCK1*, *SMC3*, *RELL2*, *COL17A1*, *ADAMTS9*, *MMP21*, *ADAMTS8*, *NAV2*, *KAZALD1*, *TGFBI*, *OTOG*, *WNT11*, *FBN2*, *TECTB*, *ENTPD1*, *FGF1*, *WNT8A*, *SPON1*, *WNT8B*	0.01
Cadherin domain^†,§^	*PCDH1*, *PCDHB8*, *FAT3*, *PCDHB3*, *PCDHB16*, *PCDHB1*, *PCDH12*, *PCDHB12*, *PCDHB10*, *DCHS1*	0.01
Cell adhesion^†,‡^	*OPCML*, *NELL1*, *SPOCK1*, *CUZD1*, *PCDHB12*, *PCDHB10*, *MEGF10*, *DCHS1*, *PCDH1*, *COL17A1*, *BARX2*, *CD44*, *FAT3*, *SORBS1*, *PCDHB16*, *TGFBI*, *ILK*, *ESAM*, *ENTPD1*, *SPON1*, *PCDHB8*, *MAGI1*, *CNTN5*, *INPPL1*, *PCDHB3*, *PCDHB1*, *CPXM2*, *PCDH12*, *CTNNA1*, *LYVE1*, *HEPACAM*, *CDON*, *ADAM12*, *NTM*, *PARVA*, *HABP2*, *FEZ1*	0.01
Cell-cell adhesion^†^	*PCDH1*, *BARX2*, *PCDHB8*, *CD44*, *FAT3*, *PCDHB16*, *PCDHB3*, *PCDHB1*, *PCDH12*, *ESAM*, *PCDHB12*, *PCDHB10*, *DCHS1*	0.04

^*^CNLs in FCAs A1 (105–124 Mb), A2 (23–37.3 Mb), D1 (21–39.4 Mb and 41.2–97 Mb), and D2 (54–88.9 Mb).

^†^GOTERM.

^‡^SP_PIR_KEYWORDS.

^§^UP_SEQ_FEATURE. Data analysed with DAVID Bioinformatics.

## Discussion

CNVs serve to identify cancer-related genomic regions^16,22,71^. To the best of our knowledge, this study represents the first analysis of CNVs influence on FMCs survival. Cats represented tubulopapillary carcinomas (group TC), and solid carcinomas and comedocarcinomas (group SC). As reported^37,72^, the group SC displayed lower survival intervals (DFS and cancer-specific OS) than the group TC. However, a subset of patients in group TC exhibited worse prognosis than others allocated in the same group. Considering this, an independent classification (St. Gallen) was applied to identify molecular subsets with distinctive biological behaviour (i.e. LA, LB *f*HER2+, LB *f*HER2−, *f*HER2+, and normal-like TN, and basal-like TN)^9,10^. Moreover, to identify factors underlying survival differences, we performed univariate and multivariate analyses including genomic characteristics explored (i.e. percentage of aberrant genomic windows, CNVs score, and presence of specific CNVs) and known malignancy indicators (e.g. histological grading, Ki-67 index, etc.).

The group TC was molecularly heterogeneous including subtypes with best and worst prognoses, which explains the outcome differences observed within the group. Conversely, the group SC mainly included highly-aberrant TN-FMCs. These results highlight the value of the St. Gallen classification to predict prognosis, especially in tubulopapillary carcinomas, in which the histological diagnosis fails to differentiate patients according to possible outcome. In line with previous studies^9,10^, molecular subtype was correlated with prognosis. We observed a negative correlation between the percentage of aberrant genomic windows and survival intervals. LA subtype tumours were the least aberrant and displayed the longest survival rates. In contrast, basal-like TN-FMCs shown the poorest survival and were the most aberrant, which provide evidence for a higher genomic instability.

To evaluate the influence of genomic instability on survival, tumours were classified into high- or low-CNVs (CNVs score). A high-CNVs score negatively influenced survival intervals in the univariate analysis. Since the recurrence of specific CNVs indicates that such regions are likely to harbour cancer-related genes^73^, we first characterised CNVs with a consistent frequency of >20% across the population. Among them, four CNGs: A3 (1–31 Mb), B4 (1–29 Mb), D4 (1–16.7 Mb), and F2 (64–82.3 Mb), and six CNLs: A2 (23–37.3 Mb, and 37.3–48 Mb), B1 (1–23 Mb), and D1 (1.1–20 Mb, 21–39.4 Mb, and 41.2–97 Mb) were associated with poor survival intervals in the univariate analysis.

CNGs associated with poor outcome harboured transcription factors *GATA3*, *GATA5*, *MLLT10*, and *TAF3* frequently amplified in HBCs^15,19,28,41–43^, EMT markers *VIM*, *LAMA5*, and *ZEB1*^21^, and several genes enriching biological processes enhancing tumour-cell migration and motility. As observed in the whole population studied, CNGs affecting basal-like TN-FMCs harboured several protein serine/threonine kinases related to cellular proliferation and motility.

CNGs negatively influencing survival intervals resemble somatic-CNGs reported in human cancer. Firstly, A3 1–31 Mb (human homologue in HSA 20q) is amplified in multiple human cancers^51–53,74^, including HBCs^19,75,76^. This region harbours all genes in an 18-potential-therapeutic-targets signature (i.e. *TPX2*, *DLGAP4*, *DSN1*, *RBL1*, *FAM83D*, *MYBL2*, *PABPC1L*, *UBE2C*, *CTSA*, *MMP9*, *SULF2*, *CSE1L*, *SALL4*, *ZNF217*, *PFDN4*, *AURKA*, *RAE1*, and *PMEPA1*) associated with poor prognosis across multiple human cancers^51^. Secondly, CNGs in the human homologue region of B4 1–29 Mb (located in HSA 10p) are associated with poor prognosis in HBCs^18,20,77^. This region harbours several HBC-related genes including *KIN*, *TAF3*, *CCDC3*, *MCM10*, *GATA3*, *PHYH* and *SEPHS1*^19,28,41^, and EMT-associated genes such as *VIM* and *ZEB1*^21^.

Thirdly, D4 1–16.7 Mb (human homologue in HSA 9p) gain is associated with a distinctive molecular subtype detected across major human cancer types, including triple-negative breast cancer (hTNBC)^57,78,79^. In this study, this aberration was observed in six patients including two normal-like TN and three basal-like TN tumours highlighting the genomic similarities between triple-negative molecular phenotypes across FMCs and HBCs. Tumours carrying this mutation are characterised by the co-gain of *PD-L1*, *PD-L2*, and *JAK2*, and might benefit from immune checkpoint targeted therapies^57,78,79^ or adjuvant therapy with *JAK2*-specific inhibitors^80,81^. Interestingly, the Jak-STAT signalling pathway was the only KEGG pathway enriched with amplified genes in the basal-like TN subtype. Finally, CNGs in F2 64–82.3 Mb (human homologue in HSA 8q) resemble a CNG reported in HBCs^18,41^, canine mammary tumours^82^, and HBC- and FMC-derived cell lines^19,83,84^. This region harbours more than 30 HBC-related genes^19,28,41^, among those *MYC*, *SCRIB*, *NDRG1*, and *PTK2* are EMT-related genes frequently amplified in human cancer^21^. Furthermore, proto-oncogene *MYC* amplification is a somatic-CNG validated by the CGC^59^.

Among CNGs detected, survival intervals remained negatively influenced by CNGs in B4 1–29 Mb and F2 64–82.3 Mb in the multivariate analysis. The influence of these aberrations on EMT-elicitation in an FMC-derived cell line was described for our group^84^. In this study, these CNGs were associated with poor outcomes and were commonly observed across the population except for the LA subtype. These results now provide evidence about their influence on FMC survival and also highlight the importance of detecting EMT-associated aberrations to predict early recurrence and reduced survival.

Besides CNGs influencing survival intervals, a CNG affecting E3 1.1–34.5 Mb (human homologue in HSA 7p) was the most common and affected all molecular subgroups except the LA subtype. This aberration is also reported in human^20^ and canine mammary tumours^82^. Genes in this region enriched the leukocyte transendothelial migration pathway. The similarities in the molecular mechanisms behind the first steps of leukocytes and neoplastic cells extravasation are well known^85,86^. Moreover, this region encompasses up to 50 genes implicated in actin cytoskeleton organization and cellular motion including *SBDS*, *ITGAM*, *PDGFA*, and *RAC1*^49^. Our findings suggest that FMC cells might use part of the molecular machinery orchestrating leukocyte extravasation to facilitate dissemination and invasion through the endothelium. Moreover, cats with tumours carrying this aberration might be a suitable model for the identification of molecular targets which suppression might prevent or reduce cellular migration and tumour dissemination. Among possible molecular targets in the region, *CLDN3* and *CLDN4* overexpression characterises invasive HBCs^87^ and *CLDN4* might be necessary for vasculogenic mimicry^88^.

On the other hand, common CNLs negatively influencing survival intervals harboured important tumour suppressors (e.g. *CSMD1*, *MTUS1*, *MSR1*, *DBC2*, and *TUSC3*) and genes enriching biological processes that would normally prevent cellular movement. Moreover, aditional genes in deleted regions enriched common deregulated metabolic pathways in cancer such as Pyruvate metabolism, PPAR signalling pathway, and N-Glycan biosynthesis^89–92^. CNLs detected included aberrations reported in HBCs and different types of human cancer. Firstly, CNLs in proximal FCA A2 (23–37.3 Mb and 37.3–48 Mb) harbouring tumour suppressors *PTPRG* and *FHIT* correspond to a fragile site in HSA 3p commonly deleted in different types of human cancer^93^ and HBC cell lines^19^. Secondly, CNLs in B1 1–23 Mb correspond to a deletion characterising poor prognosis in HBCs: CNLs at HSA 8p^23,26,27^. Similarly, among deletions detected in this study only CNLs in B1 1–23 Mb remained significantly associated with poor DFS in the multivariate analysis. Moreover, this genomic region is enriched in tumour suppressors including *CSMD1*, *FBXO25*, *ANGPT2*, *AGPAT5*, *PCM1*, *MTUS1*, *PDGFRL*, *DBC2* and *TUSC3*^19,28,41,94^.

Finally, three contiguous CNLs in FCA D1 (1–20 Mb, 21–39.4 Mb, and 41.2–97 Mb) were enriched in CAMs and tumour suppressors. Interestingly, losses in the homologue human regions (located in HSA 11q) are associated with HBC-tumourigenesis and poor survival^95,96^. The deletion or down-regulation of genes *BARX2*, *HEPACAM*, and *OPCML* included in those regions is a potential biomarker and therapeutic target in different types of human malignancies^97–101^ including HBCs^102–104^. *OPCML* and *BARX2* downregulation is associated with EMT-elicitation and poor prognosis in different types of human cancer^97,99,105^. This findings highlight the importance of this deletion as a useful biomarker for poor prognosis and its potential for the identification of therapeutic targets.

As observed in other molecular subtypes, CNLs affecting basal-like TN-FMCs harboured tumour suppressors or genes enriching biological processes normally preventing tumour dissemination. Moreover, basal-like TN-FMCs displayed additional CNLs associated with poor outcome (i.e. CNLs in A1 105–124 Mb, and D2 54–88.9 Mb) also detected in normal-like TN-FMCs. Among genes deleted, tumour suppressor *CTNNA1* (A1 105–124 Mb, human homologue in HSA 5q) is implicated in cell-cell adhesion maintainance and regulation of multiple signalling pathways in human cancer^106^. Furthermore, CNLs affecting the basal-like TN-FMCs enriched the basal cell carcinoma pathway, and the Wnt signalling pathway commonly deregulated in hTNBCs^28,77,106^. Moreover, multiple enzyme-coding genes (e.g. *LDHC*, *LDHA*, *ALDH7A1*, and *LDHAL6A*) participating in the reprogramming of metabolic pathways were deleted in this subtype.

Consistent with other studies^1,34,107^, a high-Ki-67 index remained associated with reduced cancer-specific OS in the multivariate analysis. As previously reported^1,36,38,72,108^ the EE, and the MMEE grading systems were correlated with poor outcome in the univariate analysis. Nonetheless, among the grading systems employed, the Mills-2015 system showed the highest level of agreement (66.7%) between well-differentiated tumours and low Ki-67 indexes. All systems evaluated (EE, MMEE, and Mills-2015) detected poorly-differentiated tumours efficiently, nonetheless, a minimal level of agreement among the three systems was observed (K<0.30). Furthermore, none of them revealed significant differences in survival times when comparing moderately-differentiated tumours with poorly-differentiated or with well-differentiated tumours independently.

Considering possible differences in *HER2* expression regulation between HBCs and FMCs^109–112^ and the most recent recommendations for IHC-2+ validation in HBCs^31^, we correlated *f*HER2 IHC-expression with CNGs affecting *HER2*. We identified a CNG (E1 38–44 Mb) harbouring *HER2* in seven patients (log2-ratio >0.2), four of them with a log2-ratio >0.5 including three IHC-negative (0 or 1+) and one IHC-2+ cases. In this study, two IHC-3+ tumours did not carry *HER2*-associated CNGs. In line with reports in HBCs^113,114^ and as recently reported in FMCs^110^, some IHC-3+ tumours do not display *HER2* amplifications questioning the role of this aberration as a driver mutation in FMCs. Considering the higher proportion of *f*HER2 IHC-negative cases carrying this mutation, it is possible that it might be associated with the amplification of additional cancer-related genes in the region (e.g.*CDK12*, *TOP2A*, *EIF1*, and *STAT3*) rather than inducing *f*HER2 overexpression as typically observed in HBCs.

Findings of this study in combination with reports on FMCs^109,111,115,116^ question the utility of *HER2* amplification to validate *f*HER2 IHC-overexpression. In line with previous studies^109,111,115,116^, FMCs with *f*HER2 IHC-overexpression and normal *HER2* copy-number might be a suitable model for the homologue HBC subgroup (HER2; IHC-3+/FISH-)^117^. Further studies using larger cohorts should apply the new interpretation system^31^ to evaluate the suitability of *HER2* amplifications detection to validate *f*HER2-IHC overexpression in FMCs. Besides *HER2* amplification, a higher *f*HER2 serum concentration (≥10 ng/ml) was detected in cats carrying IHC-2+ and −3+ tumours^112^. However, serum *f*HER2 levels between IHC-2+ and IHC-3+ were not statistically compared.

As *HER2*-mRNA levels positively correlate with a stronger IHC-expression on FMCs and FMC-derived cell lines without *HER2* amplification^115,116^, it is possible that as documented in HBCs^118,119^ and suggested in FMCs^110,115,116^, additional post-transcriptional or epigenetic mechanisms regulate *f*HER2 expression in FMCs. Consequently, further mechanistic studies are necessary to determine the biological significance of *f*HER2 expression levels (IHC and mRNA) in correlation with *HER2* amplification status, and to disclose possible epigenetic mechanisms inducing *f*HER2-overexpression on FMCs.

Besides somatic-CNVs affecting neoplastic cells, normal cells also have variations in the number of copies, representing a source of genomic diversification during evolution^120,121^. Those mutations usually affect smaller genomic regions than somatic-CNVs and some of them—usually referred to as germline-CNVs—are implicated in cancer inheritance^15,16,122–124^. Conversely, the abundance and typically larger size of somatic-CNVs detected in neoplastic cells imply that aneuploidy might be useful to maintain cellular proliferation and sustain tumour progression^18,96,125–127^. Considering the frequency and size of CNVs detected in this study, it is more likely that they mainly represent somatic-CNVs acquired and accumulated during clonal expansion. However, paired germline samples were not included, consequently the NGS analysis employed did not distinguish germline and somatic variants, and sequencing results may contain both findings. According to the last standards and guidelines for the interpretation and reporting of sequence variants in cancer^123^, literature review and database queries might be helpful to determine whether the CNV has been reported as a recurrent germline variant. Consequently, we compared our data with somatic-CNVs detected in HBCs^19,27,28,41–43^, somatic- and germline-CNVs validated by the CGC^59^, EMT-related genes affected by somatic-CNGs in multiple human cancer types^21^, and CNVs affecting FMC- and HBC-derived cell lines^19,84^.

As far as we know, there is only one publication characterising CNVs in cats according to different breeds (Genova *et al*.^121^), and none characterising germline-CNVs associated with FMCs predisposition. When comparing our data with those of Genova *et al*., we observed that none of the animals in that study carried CNGs affecting B4 1–29 Mb and F2 64–82.3 Mb—enriched with cancer-related genes and significantly associated with poor outcome. Interestingly, in that study, three individuals carried two small (<0.02 Mb) CNLs comprised within B1 1–23 Mb (i.e. B1 14.5 Mb, and B1 20.7 Mb). Among them, B1 20.7 Mb harbours tumour suppressor *PCM1* reported as deleted in this study and strongly associated with poor prognosis. Further studies comparing paired neoplastic-normal samples are necessary to disclose possible cancer-related germline-CNVs.

In this study, the DNA yields and 260/280 ratios of the two types of samples (FFPE and FT) were similar. However, the DNA isolated from four FFPE specimens stored for over 15 years was not suitable for library preparation. This may be associated with the effect of storage time and fixation process on DNA quality^128^. Moreover, in three samples (all LA subtype) no CNVs were detected. This might be related to the fact that DNA was isolated from all cellular subpopulations included in each sample. These samples were later excluded from CNVs analysis considering major contamination with healthy mammary tissue. This study may be biased because of a small sample size. In proportional hazards regression, the recommended number of events per variable (EPV) is 10^129^. Due to the small sample size, several independent variables were characterised with a lower EPV value. Therefore, these results need to be validated in a larger series. Additionally, the wide confidence intervals observed may also be related to small sample bias^130^.

In summary, this study points up the role of specific CNVs as useful prognostic markers in FMCs and highlight the suitability of this tool to identify possible therapeutic targets and biomarkers within the rearranged regions. The CNVs landscape of FMCs included multiple rearranged genomic regions. Importantly, the amount and frequency of CNVs varied among molecular subtypes evaluated and were associated with prognosis. Moreover, some aberrations were predominantly observed in TN-FMCs. Among rearrangements detected, CNGs in A3 (1–31 Mb), B4 (1–29 Mb), D4 (1–16.7 Mb), and F2 (64–82.3 Mb) were common and negatively influenced survival intervals. Moreover, those regions harbour several oncogenes and cancer-related genes enhancing cellular movement and migration-related pathways. Among CNGs, those affecting FCAs B4 and F2 negatively influenced DFS and cancer-specific OS in the multivariate analysis and included multiple EMT-associated genes. On the other hand, common CNLs in FCAs A2 (23–37.3 Mb and 37.3–48 Mb), B1 (1–23 Mb), and D1 (1.1–20 Mb, 21–39.4 Mb, and 41.2–97 Mb) were enriched with tumour repressors and encompassed genes involved in biological processes that would normally prevent cellular movement and migration. Among them, CNLs in B1 (1–23 Mb) remained associated with poor DFS in the multivariate analysis. Further efforts are necessary to sequence a larger series of FMC genomes, characterise somatic CNVs significantly associated with reduced survival, disclose possible germline-CNVs comprised within extensive rearrangements detected in this study, and investigate the suitability of cancer-related genes reported here as possible biomarkers and therapeutic targets on FMCs.

## Methods

### Animals

Thirty-three female cats surgically treated for FMC at the Small Animal Clinic of the University of Veterinary Medicine Hannover from 2000 to 2016 were enrolled in this study. Clinical history and reproductive status were obtained. Only cats, for which thoracic radiographs to investigate the presence of metastases at diagnosis and during the follow-up period were available, were included in this study. Patients were clinically staged using the modified World Health Organization (WHO) system^8^. Due to the previously reported reduced survival of cats with solid carcinomas (8 months) compared to tubulopapillary carcinomas (36 months)^1,72^, patients were grouped based on biological behaviour and morphology of the tumours into two categories (TC and SC). The group TC included tubulopapillary carcinomas, and the group SC comprised both solid carcinomas and comedocarcinomas representing six molecular subtypes. All cats underwent unilateral chain mastectomy, including resection of involved lymph nodes. The patients were followed up for a two-year post-operative period or until death. Long-term follow-up was obtained by clinical records analysis and telephone interviews with the owners.

### Tissue samples

FFPE and/or FT tumour samples from 31 cats were retrospectively collected for histopathological examination and DNA isolation. In those cases, the FFPE specimens were retrieved from the archives of the Department of Pathology, University of Veterinary Medicine Hannover. The FT samples from patients retrospectively included were retrieved from the frozen tissue bank of the Small Animal Clinic, University of Veterinary Medicine Hannover. In two cases, FFPE and FT samples were prospectively collected. All samples were collected for diagnostic purposes during the medically necessary surgery after owner’s written approval. Consequently, this study was not an animal experiment according to the German Animal Welfare Act and ethical approval was not required.

### Histopathological examination

Paraffin sections (4 µm) of the tumour samples were stained with haematoxylin eosin (H&E) for histopathological evaluation. The samples were examined under light microscopy and the morphological diagnosis was performed following the WHO classification^7^. Histological grading of the tumours was performed using the EE system^35,36^. Additionally, we applied the MMEE grading system, and the feline-specific grading system proposed by Mills *et al*. (Mills-2015)^37^. For each case of our cohort, the cumulative number of mitoses was determined in 10 consecutive fields in the most mitotically active area with a field diameter of 0.575 mm (40x objective). The mitotic cut-offs for the EE and MMEE grading systems in our cohort were adapted according to the field diameter (0.51 in Castagnaro *et al*.^36^, 0.53 in Mills *et al*.^37^, and 0.575 in our study). For the Mills-2015 system, as suggested by Dagher *et al*.^38^, we determined the mitotic count cut-off by using receiver-operating characteristic (ROC) analysis for the study period OS-cancer specific survival rate of the cats included in this study. Therefore the mitotic count cut-off used in this study (≥35 mitoses in 10 high-power fields) was different from that proposed by Mills *et al*.^37^ and used by Dagher *et al*.^38^ (>62, and ≥33 mitoses in 10 high-power fields; respectively).Complete surgical excision and lymph node metastasis were histopathologically confirmed in all cases.

### Immunohistochemical examination

The expression of ER, PR, HER2, CK5/6, and proliferation marker Ki-67 was evaluated by the avidin-biotin complex technique as previously described^131^. No animal was killed for the generation of positive controls, all tissue specimens used as positive controls for this study were sent to the Department of Pathology for diagnosis. Negative controls included normal serum and isotype control antibodies (details in Supplementary Table 8).

Tumours were classified into six subtypes (LA, LB *f*HER2+, LB *f*HER2−, *f*HER2+, normal-like TN and basal-like TN) on the basis of their ER, PR, *f*HER2, CK5/6, and Ki-67 expression as described elsewhere^9,10,132^, details in Supplementary Table 9. Tumours with an Allred score (Supplementary Table 10) equal to or higher than three were considered positive for ER or PR^10,133,134^. *f*HER2 expression was evaluated as described elsewhere^10,11,31,109,135^, Supplementary Table 11. Tumours scored as positive (3+) by IHC were considered as *f*HER2-positive for the St. Gallen classification. According to the last recommendations of the American Society of Clinical Oncology (ASCO) and the College of American Pathologists (CAP) for the validation of cases scored as HER2-2+ by IHC in HBC^31^, and in agreement with the high concordance reported between *HER2*-amplifications detected using FISH/ISH and CNGs detected by different high-resolution sequencing methods to validate HER2 status in human breast cancer (HBC)^136–138^, we correlated *f*HER2 IHC-based expression with CNGs affecting *HER2* gene coding region (FCA E1 40,780,250–40,804,241 Mb). As reported elsewhere^136–139^, log2-ratio threshold for *HER2* amplification was set at a higher amplitude (>0.5) for *f*HER2+ tumours detection to better show gene-level CNV events^140^. Accordingly, cases scored as 2+ with CNGs (log2-copy-number-ratio >0.5^141^) in FCA E1 harbouring *HER2* were considered as *f*HER2-positive.

To identify tumours expressing basal markers, we evaluated the expression of CK5/6. Tumours were considered positive if more than 1% of the tumour cells showed positive cytoplasmic labelling^142^. The Ki-67 proliferation index was calculated as the percentage of positively stained tumour cell nuclei in 1000 tumour cells. Five to six microphotographs (40X) were randomly taken and analysed using Image J (Open Source Software, version 1.8.0; National Institutes of Health, Bethesda, MD, USA). Moreover, the Ki-67 index was considered as high when the number of positive nuclei was higher or equal than 14%^34^.

### DNA isolation

FFPE samples: four 10-µm-thick sections were sliced using a microtome (pfm Slide 2003, pfm medical ag). Afterwards, the sections were deparaffinised and the nucleic acids were isolated with the AllPrep DNA/RNA FFPE kit (QIAGEN) following the manufacturer’s instructions. FT samples: the frozen tissue was previously homogenised using a TissueLyser (II—5 mm stainless steel bead, QIAGEN GmbH, Hilden, Germany). DNA was isolated using the AllPrep DNA/RNA Mini Kit (QIAGEN) following the manufacturer’s instructions. The DNA yields were determined with the Synergy 2 microplate reader (BioTek) and the purity of each sample was determined by calculating the 260/280 ratio. Samples were stored at −80 °C until use.

### CNV analysis

DNA sequencing and CNVs analysis were performed according to the methodology described by Granados-Soler *et al*.^84^. 100-500 ng DNA was sheared to a size of 200–500 bp by ultrasound using a Covaris S2 focused ultrasonicator and sequencing libraries were prepared using the NEBNext UltraTM II DNA Library Preparation Kit for Illumina® (New England Biolabs). Paired-end sequencing (38/37bp) was conducted on an Illumina NextSeq500 according to manufacturer’s instructions. On average 36.3 Mb (SD: 5.5 Mb) reads were generated (Supplementary Table 5). Of those an average of 29.6 Mb (SD: 6 Mb) reads was mapped to the feline reference genome FelCat6.2 using BWA.

As recommended by Genova *et al*.^121^, two different programs were used to call large size aneuploidies by depth of coverage analysis and results were compared to reduce false-positive calls. First, we applied two different programs to generate log2-copy-number-ratios for each cancer sample: 1. CNV-seq^39^ and 2. CNVKit^40^. For both analyses, a fixed window size of 2 Mb was used and tumour samples were compared against two normal reference samples obtained from feline healthy mammary frozen tissue. Control tissue was collected from two recently euthanised (for medical reasons) intact female Domestic Shorthair cats (five- and seven-years-old) with no pathologies of the mammary gland. DNA was isolated and stored using the same methodology employed for neoplastic FT samples (described above).

The log2-ratios obtained by the CNVKit method “WGS” were corrected for the highly aneuploid tumour samples using the method “centre mode” to shift the true neutral regions closer to zero. Second, the results (log2-ratios) of both algorithms were smoothed using a circular binary segmentation algorithm implemented in the program “Copynumber”^143^. For the CNV-seq data a gamma and kmin of 20 were used, while a gamma of 10 was used for the CNVKit data, because different from CNV-seq, CNVKit does not implement a sliding window binning approach resulting in only half the number of total bins. Third, from the segmented output only regions with smoothed-log2-copy-number-ratios of >0.2 or <−0.2 and >0.2 or <−0.1 for the CNV-seq and CNVKit data, respectively, were scored as significantly aberrant. Significant CNV-seq regions with >80% overlap to a significant CNVKit region were scored as validated. To refine the region boundaries, the start position of a validated CNV region was set to the maximum of both start positions and the stop position was set to the minimum of both stop positions. The validated and refined CNV regions of all samples were used for generating the frequency plots with the R package GenVisR for data generation^144^, and the Circos program^145^ for plotting.

Common CNG/CNL segments were defined by frequency-changepoints, i.e. every region with a consistent frequency of >20% gains or losses across the population was defined as a common CNG or CNL segment that was used as independent variable in multivariate regression testing. Moreover, structural rearrangements detected (CNGs and CNLs) were compared with somatic-CNVs detected in HBCs^19,27,28,41–43^, somatic- and germline-CNVs validated by the CGC^59^, EMT-related genes affected by somatic-CNGs in multiple human cancer types^21^, CNVs affecting FMC- and HBC-derived cell lines^19,84^, and additional cancer-associated genes previously reported on FMCs.

Genes in regions affected by CNVs were identified in the reference genome sequences provided by the UCSC Genome Bioinformatics Site (http://genome.ucsc.edu/). Additionally, to determine enriched biological terms, lists of the genes harboured in genomic regions most commonly affected by CNVs, and those correlated with poor outcome (reduced DFS and/or cancer-specific OS) were uploaded to the DAVID Bioinformatics server (http://david.abcc.ncifcrf.gov), which was employed for functional annotation clustering and KEGG pathway analysis^146,147^.

### Statistical analysis

The statistical software SPSS (IBM SPSS Statistics for Windows, Version 23.0. Armonk, NY, USA) was employed to perform all statistical analyses. For all statistical analyses, a *p* ≤ 0.05 was considered significant. Epidemiological, clinical, and histopathological variables included were age, breed, reproductive status, tumour size, lymph node metastasis (confirmed by histopathology), distant metastasis, modified WHO clinical stage^8^, and histological grading (EE, MMEE, and Mills-2015). CNVs, CNGs, and CNLs were categorised (CNVs score) into high or low according to whether the percentage of affected bins was lower or greater than their respective median values. Presence or absence of specific CNVs (CNGs and/or CNLs) affecting >20% of the sample size was included as categorical variables. Local recurrence, distant metastases, and death were considered as follow-up variables. Descriptive statistics of patients allocated in histological categories TC and SC regarding all epidemiological, clinical, histopathological and follow-up variables were performed. Percent agreement calculation was used to compare histological grades assigned (I, II and III) using the three systems employed and Ki-67 scores (low or high^34^). Moreover, the level of agreement using the three different histological grading systems employed was compared using Cohen's Kappa test^148^. DFS and cancer-specific OS were defined as months from surgery to tumour recurrence (local or distant), and to death, respectively. Cats were censored from DFS analysis at death without recurrence. Animals alive at the end of the study period (24 months) and animals that died due to non-tumour related causes were censored from cancer-specific OS analysis. Differences regarding all epidemiological, clinical, histopathological and molecular characteristics were assessed using T-Test. Univariate Kaplan-Meier log-rank analyses were independently applied to determine whether histologic diagnosis, molecular subtype, CNVs score, presence or absence of specific CNVs and all additional epidemiological, clinical, and histopathological variables were associated with reduced DFS and cancer-specific OS. A multivariate Forward Stepwise Cox proportional hazards regression analysis was performed to assess the influence of independent variables on dependent follow-up variables (DFS and OS), the variable entry and retention criteria were set at 0.25 and 0.1, respectively^149^.

## Supplementary information

Supporting Information.

## Data Availability

The datasets generated and/or analysed during the current study are available from the corresponding author on reasonable request.

## References

[1] Zappulli V (2015). Prognostic Evaluation of Feline Mammary Carcinomas: A Review of the Literature. Veterinary Pathol..

[2] Hahn KA, Bravo L, Avenell JS (1994). Feline breast carcinoma as a pathologic and therapeutic model for human breast cancer. vivo.

[3] Hahn KA, Adams WH (1977). Feline mammary neoplasia: biological behavior, diagnosis, and treatment. Feline Pract..

[4] Gimenez F, Hecht S, Craig LE, Legendre AM (2010). Early detection, aggressive therapy: optimizing the management of feline mammary masses. J. Feline Med. Surg..

[5] Hayden DW, Nielsen SW (1971). Feline mammary tumours. J. Small Anim. Pract..

[6] Morris J (2013). Mammary tumours in the cat: size matters, so early intervention saves lives. J. Feline Med. Surg..

[7] Misdorp, W., Else, R. W., Hellmen, E. & Lipscomb, T. P. *Histological classification of mammary tumors of the dog and cat*. 2nd edn, (1999).

[8] McNeill CJ (2009). Evaluation of adjuvant doxorubicin-based chemotherapy for the treatment of feline mammary carcinoma. J. Vet. Intern. Med..

[9] Soares M, Correia J, Peleteiro MC, Ferreira F (2016). St Gallen molecular subtypes in feline mammary carcinoma and paired metastases-disease progression and clinical implications from a 3-year follow-up study. Tumour Biol..

[10] Soares M (2016). Molecular based subtyping of feline mammary carcinomas and clinicopathological characterization. Breast.

[11] Park S (2012). Characteristics and outcomes according to molecular subtypes of breast cancer as classified by a panel of four biomarkers using immunohistochemistry. Breast.

[12] Badve S (2011). Basal-like and triple-negative breast cancers: a critical review with an emphasis on the implications for pathologists and oncologists. Mod. Pathol..

[13] Cejalvo JM (2017). Intrinsic Subtypes and Gene Expression Profiles in Primary and Metastatic Breast Cancer. Cancer Res..

[14] Vuong D, Simpson PT, Green B, Cummings MC, Lakhani SR (2014). Molecular classification of breast cancer. Virchows Arch..

[15] Horpaopan S (2015). Genome-wide CNV analysis in 221 unrelated patients and targeted high-throughput sequencing reveal novel causative candidate genes for colorectal adenomatous polyposis. Int. J. Cancer.

[16] Shlien A, Malkin D (2009). Copy number variations and cancer. Genome Med..

[17] Singh RR (2014). Clinical massively parallel next-generation sequencing analysis of 409 cancer-related genes for mutations and copy number variations in solid tumours. Br. J. Cancer.

[18] Gao R (2016). Punctuated copy number evolution and clonal stasis in triple-negative breast cancer. Nat. Genet..

[19] eSaito S, Morita K, Hirano T (2009). High frequency of common DNA copy number abnormalities detected by bacterial artificial chromosome array comparative genomic hybridization in 24 breast cancer cell lines. Hum. Cell.

[20] Bergamaschi A (2006). Distinct patterns of DNA copy number alteration are associated with different clinicopathological features and gene-expression subtypes of breast cancer. Genes. Chromosomes Cancer.

[21] Zhao M, Liu Y, Qu H (2016). Expression of epithelial-mesenchymal transition-related genes increases with copy number in multiple cancer types. Oncotarget.

[22] Zack TI (2013). Pan-cancer patterns of somatic copy number alteration. Nat. Genet..

[23] Lebok P (2015). 8p deletion is strongly linked to poor prognosis in breast cancer. Cancer Biol. Ther..

[24] Iddawela M (2017). Integrative analysis of copy number and gene expression in breast cancer using formalin-fixed paraffin-embedded core biopsy tissue: a feasibility study. BMC Genomics.

[25] Stephens PJ (2009). Complex landscapes of somatic rearrangement in human breast cancer genomes. Nat..

[26] Moelans CB, van Maldegem CMG, van der Wall E, van Diest PJ (2018). Copy number changes at 8p11-12 predict adverse clinical outcome and chemo- and radiotherapy response in breast cancer. Oncotarget.

[27] Cai Y (2016). Loss of Chromosome 8p Governs Tumor Progression and Drug Response by Altering Lipid Metabolism. Cancer Cell.

[28] Ellis MJ (2012). Whole-genome analysis informs breast cancer response to aromatase inhibition. Nat..

[29] Knight JF (2018). KIBRA (WWC1) Is a Metastasis Suppressor Gene Affected by Chromosome 5q Loss in Triple-Negative Breast Cancer. Cell Rep..

[30] Ross JS (2015). Genomic Profiling of Advanced-Stage, Metaplastic Breast Carcinoma by Next-Generation Sequencing Reveals Frequent, Targetable Genomic Abnormalities and Potential New Treatment Options. Arch. Pathol. Laboratory Med..

[31] Wolff AC (2018). Human Epidermal Growth Factor Receptor 2 Testing in Breast Cancer: American Society of Clinical Oncology/College of American Pathologists Clinical Practice Guideline Focused Update. Arch. Pathol. Lab. Med..

[32] Ferrari A (2016). A whole-genome sequence and transcriptome perspective on HER2-positive breast cancers. Nat. Commun..

[33] Kim JY (2019). Molecular alterations and poziotinib efficacy, a pan-HER inhibitor, in human epidermal growth factor receptor 2 (HER2)-positive breast cancers: Combined exploratory biomarker analysis from a phase II clinical trial of poziotinib for refractory HER2-positive breast cancer patients. Int. J. Cancer.

[34] Soares M (2016). Ki-67 as a Prognostic Factor in Feline Mammary Carcinoma: What Is the Optimal Cutoff Value?. Vet. Pathol..

[35] Elston CW, Ellis IO (1991). Pathological prognostic factors in breast cancer. I. The value of histological grade in breast cancer: experience from a large study with long-term follow-up. Histopathology.

[36] Castagnaro M (1998). Tumour grading and the one-year post-surgical prognosis in feline mammary carcinomas. J. Comp. Pathol..

[37] Mills SW (2015). Prognostic value of histologic grading for feline mammary carcinoma: a retrospective survival analysis. Vet. Pathol..

[38] Dagher E, Abadie J, Loussouarn D, Campone M, Nguyen F (2019). Feline Invasive Mammary Carcinomas: Prognostic Value of Histological Grading. Vet. Pathol..

[39] Xie C, Tammi MT (2009). CNV-seq, a new method to detect copy number variation using high-throughput sequencing. BMC Bioinforma..

[40] Talevich E, Shain AH, Botton T, Bastian BC (2016). CNVkit: Genome-Wide Copy Number Detection and Visualization from Targeted DNA Sequencing. PLoS Comput. Biol..

[41] Haverty PM (2008). High-resolution genomic and expression analyses of copy number alterations in breast tumors. Genes. Chromosomes Cancer.

[42] Nik-Zainal S (2012). Mutational Processes Molding the Genomes of 21 Breast Cancers. Cell.

[43] Pongor L (2015). A genome-wide approach to link genotype to clinical outcome by utilizing next generation sequencing and gene chip data of 6,697 breast cancer patients. Genome Med..

[44] Bai M (2011). ARFGAP1 promotes AP-2-dependent endocytosis. Nat. Cell Biol..

[45] Mellman I, Yarden Y (2013). Endocytosis and cancer. Cold Spring Harb. Perspect. Biol..

[46] Jones MC, Caswell PT, Norman JC (2006). Endocytic recycling pathways: emerging regulators of cell migration. Curr. Opin. Cell Biol..

[47] Durand N (2018). The phosphorylation status of PIP5K1C at serine 448 can be predictive for invasive ductal carcinoma of the breast. Oncotarget.

[48] Inoue H, Ha VL, Prekeris R, Randazzo PA (2008). Arf GTPase-activating protein ASAP1 interacts with Rab11 effector FIP3 and regulates pericentrosomal localization of transferrin receptor-positive recycling endosome. Mol. Biol. Cell.

[49] Stelzer G (2016). The GeneCards Suite: From Gene Data Mining to Disease Genome Sequence Analyses. Curr. Protoc. Bioinforma..

[50] Byerly J, Halstead-Nussloch G, Ito K, Katsyv I, Irie HY (2016). PRKCQ promotes oncogenic growth and anoikis resistance of a subset of triple-negative breast cancer cells. Breast Cancer Res..

[51] Snijders AM, Mao JH (2016). Multi-omics approach to infer cancer therapeutic targets on chromosome 20q across tumor types. Adv. Mod. Oncol. Res..

[52] Wilting SM (2006). Increased gene copy numbers at chromosome 20q are frequent in both squamous cell carcinomas and adenocarcinomas of the cervix. J. Pathol..

[53] Bouchard D, Morisset D, Bourbonnais Y, Tremblay GM (2006). Proteins with whey-acidic-protein motifs and cancer. Lancet Oncol..

[54] Leblanc R, Sahay D, Houssin A, Machuca-Gayet I, Peyruchaud O (2018). Autotaxin-beta interaction with the cell surface via syndecan-4 impacts on cancer cell proliferation and metastasis. Oncotarget.

[55] Rahimi Azadeh, Sedighi Rina, Emadi‐Baygi Modjtaba, Honardoost Mohammad‐Amin, Mowla Seyed‐Javad, Khanahmad Hossein, Nikpour Parvaneh (2019). Bioinformatics prediction and experimental validation of a novel microRNA: hsa‐miR‐B43 within human CDH4 gene with a potential metastasis‐related function in breast cancer. Journal of Cellular Biochemistry.

[56] Hollern DP (2019). E2F1 Drives Breast Cancer Metastasis by Regulating the Target Gene FGF13 and Altering Cell Migration. Sci. Rep..

[57] Budczies J, Denkert C, Győrffy B, Schirmacher P, Stenzinger A (2017). Chromosome 9p copy number gains involving PD-L1 are associated with a specific proliferation and immune-modulating gene expression program active across major cancer types. BMC Med. Genomics.

[58] Sarhadi VK (2014). Copy Number Alterations and Neoplasia-Specific Mutations in MELK, PDCD1LG2, TLN1, and PAX5 at 9p in Different Neoplasias. Gene Chromosome Canc.

[59] Forbes SA (2015). COSMIC: exploring the world's knowledge of somatic mutations in human cancer. Nucleic Acids Res..

[60] Li M (2014). ASAP1 mediates the invasive phenotype of human laryngeal squamous cell carcinoma to affect survival prognosis. Oncol. Rep..

[61] Muller T (2010). ASAP1 promotes tumor cell motility and invasiveness, stimulates metastasis formation *in vivo*, and correlates with poor survival in colorectal cancer patients. Oncogene.

[62] Lu S (2016). Overexpression of HOXC8 is Associated With Poor Prognosis in Epithelial Ovarian Cancer. Reprod. Sci..

[63] Lu L (2017). Identification of circular RNAs as a promising new class of diagnostic biomarkers for human breast cancer. Oncotarget.

[64] Ma J (2013). Role of activated Rac1/Cdc42 in mediating endothelial cell proliferation and tumor angiogenesis in breast cancer. PLoS One.

[65] Morrison Joly M (2017). Two distinct mTORC2-dependent pathways converge on Rac1 to drive breast cancer metastasis. Breast Cancer Res..

[66] Hassan BB (2017). Feline Mammary Cancer. Vet. Pathol..

[67] Sang Y (2015). TEL2 suppresses metastasis by down-regulating SERPINE1 in nasopharyngeal carcinoma. Oncotarget.

[68] Pavon MA (2016). uPA/uPAR and SERPINE1 in head and neck cancer: role in tumor resistance, metastasis, prognosis and therapy. Oncotarget.

[69] Azimi I, Petersen RM, Thompson EW, Roberts-Thomson SJ, Monteith GR (2017). Hypoxia-induced reactive oxygen species mediate N-cadherin and SERPINE1 expression, EGFR signalling and motility in MDA-MB-468 breast cancer cells. Sci. Rep..

[70] Yuan J (2018). Tumor suppressor candidate 3: A novel grading tool and predictor of clinical malignancy in human gliomas. Oncol. Lett..

[71] Beroukhim R (2010). The landscape of somatic copy-number alteration across human cancers. Nat..

[72] Seixas F, Palmeira C, Pires MA, Bento MJ, Lopes C (2011). Grade is an independent prognostic factor for feline mammary carcinomas: A clinicopathological and survival analysis. Veterinary J..

[73] Korkola J, Gray JW (2010). Breast cancer genomes–form and function. Curr. Opin. Genet. Dev..

[74] Chen Y (2016). WAP four-disulfide core domain protein 2 gene(WFDC2) is a target of estrogen in ovarian cancer cells. J. Ovarian Res..

[75] Tanner MM (1995). Amplification of chromosomal region 20q13 in invasive breast cancer: prognostic implications. Clin. Cancer Res..

[76] Fridlyand J (2006). Breast tumor copy number aberration phenotypes and genomic instability. BMC Cancer.

[77] Cancer Genome Atlas, N. (2012). Comprehensive molecular portraits of human breast tumours. Nat..

[78] Barrett MT (2015). Genomic amplification of 9p24.1 targeting JAK2, PD-L1, and PD-L2 is enriched in high-risk triple negative breast cancer. Oncotarget.

[79] Balko JM (2016). Triple-negative breast cancers with amplification of JAK2 at the 9p24 locus demonstrate JAK2-specific dependence. Sci. Transl. Med..

[80] Hernandez Boluda JC, Gomez M, Perez A (2016). JAK2 inhibitors. Med. Clin..

[81] Lin TE (2018). A Novel Selective JAK2 Inhibitor Identified Using Pharmacological Interactions. Front. Pharmacol..

[82] Beck J (2013). Genome aberrations in canine mammary carcinomas and their detection in cell-free plasma DNA. PLoS One.

[83] Jonsson G (2007). High-resolution genomic profiles of breast cancer cell lines assessed by tiling BAC array comparative genomic hybridization. Genes. Chromosomes Cancer.

[84] Granados-Soler JL (2018). TiHo-0906: a new feline mammary cancer cell line with molecular, morphological, and immunocytological characteristics of epithelial to mesenchymal transition. Sci. Rep..

[85] Liu H (2018). PRDM4 mediates YAP-induced cell invasion by activating leukocyte-specific integrin beta2 expression. EMBO Rep..

[86] Strell C, Entschladen F (2008). Extravasation of leukocytes in comparison to tumor cells. Cell Commun. Signal..

[87] Katayama A (2017). Expression patterns of claudins in patients with triple-negative breast cancer are associated with nodal metastasis and worse outcome. Pathol. Int..

[88] Cui YF (2015). Claudin-4 is required for vasculogenic mimicry formation in human breast cancer cells. Oncotarget.

[89] Harrelson JP, Lee MW (2016). Expanding the view of breast cancer metabolism: Promising molecular targets and therapeutic opportunities. Pharmacol. Ther..

[90] Durinck S (2015). Spectrum of diverse genomic alterations define non-clear cell renal carcinoma subtypes. Nat. Genet..

[91] Furuta E, Okuda H, Kobayashi A, Watabe K (2011). Metabolic genes in cancer: their roles in tumor progression and clinical implications. Biochim. Biophys. Acta.

[92] Vaiana CA, Kurcon T, Mahal LK (2016). MicroRNA-424 Predicts a Role for beta-1,4 Branched Glycosylation in Cell Cycle Progression. J. Biol. Chem..

[93] McAvoy S (2007). Non-random inactivation of large common fragile site genes in different cancers. Cytogenet. Genome Res..

[94] Sun PC (2001). Transcript map of the 8p23 putative tumor suppressor region. Genomics.

[95] Koreth J, Bakkenist CJ, McGee JO (1997). Allelic deletions at chromosome 11q22-q23.1 and 11q25-qterm are frequent in sporadic breast but not colorectal cancers. Oncogene.

[96] Litviakov NV (2016). Deletions of multidrug resistance gene loci in breast cancer leads to the down-regulation of its expression and predict tumor response to neoadjuvant chemotherapy. Oncotarget.

[97] Mi Y (2016). Downregulation of homeobox gene Barx2 increases gastric cancer proliferation and metastasis and predicts poor patient outcomes. Oncotarget.

[98] Abdel-Rahman WM (2016). The Role of Chromosomal Instability and Epigenetics in Colorectal Cancers Lacking beta-Catenin/TCF Regulated Transcription. Gastroenterol. Res. Pract..

[99] Li CH (2015). OPCML is frequently methylated in human colorectal cancer and its restored expression reverses EMT via downregulation of smad signaling. Am. J. Cancer Res..

[100] Antony J (2018). The tumour suppressor OPCML promotes AXL inactivation by the phosphatase PTPRG in ovarian cancer. EMBO Rep..

[101] Wu M, Moh MC, Schwarz H (2016). HepaCAM associates with connexin 43 and enhances its localization in cellular junctions. Sci. Rep..

[102] Schulten HJ (2017). Comprehensive molecular biomarker identification in breast cancer brain metastases. J. Transl. Med..

[103] Zanini E (2017). The Tumor-Suppressor Protein OPCML Potentiates Anti-EGFR- and Anti-HER2-Targeted Therapy in HER2-Positive Ovarian and Breast Cancer. Mol. Cancer Ther..

[104] Moradi Marjaneh, M. *et al*. High-throughput allelic expression imbalance analyses identify candidate breast cancer risk genes Mahdi. *bioRxiv*, 1–21, 10.1101/521013 (2019).

[105] Zhang Y (2015). Low expression of BARX2 in human primary hepatocellular carcinoma correlates with metastasis and predicts poor prognosis. Hepatol. Res..

[106] Sun Y, Zhang J, Ma L (2014). alpha-catenin. A tumor suppressor beyond adherens junctions. Cell Cycle.

[107] Castagnaro M (1998). Ki-67 index as indicator of the post-surgical prognosis in feline mammary carcinomas. Res. Veterinary Sci..

[108] Millanta F, Citi S, Della Santa D, Porciani M, Poli A (2006). COX-2 expression in canine and feline invasive mammary carcinomas: correlation with clinicopathological features and prognostic molecular markers. Breast Cancer Res. Treat..

[109] Soares M (2013). Feline HER2 protein expression levels and gene status in feline mammary carcinoma: optimization of immunohistochemistry (IHC) and *in situ* hybridization (ISH) techniques. Microsc. Microanal..

[110] Muscatello LV (2019). HER2 Amplification Status in Feline Mammary Carcinoma: A Tissue Microarray-Fluorescence *In Situ* Hydridization-Based Study. Vet. Pathol..

[111] Ordas J, Millan Y, Dios R, Reymundo C, de Las Mulas JM (2007). Proto-oncogene HER-2 in normal, dysplastic and tumorous feline mammary glands: an immunohistochemical and chromogenic *in situ* hybridization study. BMC Cancer.

[112] Soares M (2016). Serum HER2 levels are increased in cats with mammary carcinomas and predict tissue HER2 status. Oncotarget.

[113] Luoh SW (2013). HER-2 gene amplification in human breast cancer without concurrent HER-2 over-expression. Springerplus.

[114] Petroni S (2016). FISH testing of HER2 immunohistochemistry 1+ invasive breast cancer with unfavorable characteristics. Oncol. Lett..

[115] De Maria R (2005). Spontaneous feline mammary carcinoma is a model of HER2 overexpressing poor prognosis human breast cancer. Cancer Res..

[116] Santos S (2013). ERBB2 in Cat Mammary Neoplasias Disclosed a Positive Correlation between RNA and Protein Low Expression Levels: A Model for erbB-2 Negative Human Breast Cancer. PLoS One.

[117] Koo JS, Jung W, Yang WI (2011). HER-2 protein overexpressing breast cancer without gene amplification shows higher hormone receptor expression than HER-2 protein overexpressing breast cancer with gene amplification. Int. J. Surg. Pathol..

[118] Liu Q (2018). A novel HER2 gene body enhancer contributes to HER2 expression. Oncogene.

[119] Singla H (2017). Recent advances in HER2 positive breast cancer epigenetics: Susceptibility and therapeutic strategies. Eur. J. Med. Chem..

[120] Perry GH (2008). The evolutionary significance of copy number variation in the human genome. Cytogenet. Genome Res..

[121] Genova F (2018). First genome-wide CNV mapping in FELIS CATUS using next generation sequencing data. BMC Genomics.

[122] Huang KL (2018). Pathogenic Germline Variants in 10,389 Adult Cancers. Cell.

[123] Li, M. M. *et al*. in *A Joint Consensus Recommendation of the Association for Molecular Pathology*, *American Society of Clinical Oncology*, *and College of American Pathologists* Vol. **19**, 123–125 (2017). 10.1016/j.jmoldx.2016.10.002 PMC5707196 27993330

[124] Knouse KA, Wu J, Amon A (2016). Assessment of megabase-scale somatic copy number variation using single-cell sequencing. Genome Res..

[125] Wang K (2007). PennCNV: an integrated hidden Markov model designed for high-resolution copy number variation detection in whole-genome SNP genotyping data. Genome Res..

[126] Ozery-Flato M, Linhart C, Trakhtenbrot L, Izraeli S, Shamir R (2011). Large-scale analysis of chromosomal aberrations in cancer karyotypes reveals two distinct paths to aneuploidy. Genome Biol..

[127] Stevens KN (2011). Evaluation of associations between common variation in mitotic regulatory pathways and risk of overall and high grade breast cancer. Breast Cancer Res. Treat..

[128] Hedegaard J (2014). Next-generation sequencing of RNA and DNA isolated from paired fresh-frozen and formalin-fixed paraffin-embedded samples of human cancer and normal tissue. PLoS One.

[129] Peduzzi P, Concato J, Feinstein AR, Holford TR (1995). Importance of events per independent variable in proportional hazards regression analysis. II. Accuracy Precis. Regres. estimates. J. Clin. Epidemiol..

[130] Greenland S, Mansournia MA, Altman DG (2016). Sparse data bias: a problem hiding in plain sight. BMJ.

[131] Hammer SC (2016). Longitudinal Claudin Gene Expression Analyses in Canine Mammary Tissues and Thereof Derived Primary Cultures and Cell Lines. Int. J. Mol. Sci..

[132] Goldhirsch A (2013). Personalizing the treatment of women with early breast cancer: highlights of the St Gallen International Expert Consensus on the Primary Therapy of Early Breast Cancer 2013. Ann. Oncol..

[133] Soares M, Correia J, Murta A, Ferreira F (2013). Immunophenotyping of primary and metastatic lesions in feline mammary tumors - are they equal?. Microscopy Microanalysis.

[134] Mohsin SK (2004). Progesterone receptor by immunohistochemistry and clinical outcome in breast cancer: a validation study. Mod. Pathol..

[135] Wolff AC (2013). Recommendations for human epidermal growth factor receptor 2 testing in breast cancer: American Society of Clinical Oncology/College of American Pathologists clinical practice guideline update. J. Clin. Oncol..

[136] Biermann J (2018). Clonal relatedness in tumour pairs of breast cancer patients. Breast Cancer Res..

[137] Biermann, J. *Tumour evolution and novel biomarkers in breast cancer* PhD thesis, University of Gothenburg. Sahlgrenska Academy., (2019).

[138] Goldhirsch A (2011). Strategies for subtypes–dealing with the diversity of breast cancer: highlights of the St. Gallen International Expert Consensus on the Primary Therapy of Early Breast Cancer 2011. Ann. Oncol..

[139] Staaf J (2010). High-resolution genomic and expression analyses of copy number alterations in HER2-amplified breast cancer. Breast Cancer Res..

[140] Mermel CH (2011). GISTIC2.0 facilitates sensitive and confident localization of the targets of focal somatic copy-number alteration in human cancers. Genome Biol..

[141] Biermann J (2018). Clonal relatedness in tumour pairs of breast cancer patients. Breast Cancer Res..

[142] Brunetti B (2013). Molecular phenotype in mammary tumours of queens: correlation between primary tumour and lymph node metastasis. J. Comp. Pathol..

[143] Nilsen, G., Liestol, K. & Lingjaerde, O. C. Copynumber: Segmentation of single- and multi-track copy number data by penalized least squares regression. R package version 1.22.0. (2013).

[144] Skidmore ZL (2016). GenVisR: Genomic Visualizations in R. Bioinforma..

[145] Krzywinski M (2009). Circos: an information aesthetic for comparative genomics. Genome Res..

[146] Huang DW, Sherman BT, Lempicki RA (2008). Systematic and integrative analysis of large gene lists using DAVID bioinformatics resources. Nat. Protoc..

[147] Huang da W, Sherman BT, Lempicki RA (2009). Bioinformatics enrichment tools: paths toward the comprehensive functional analysis of large gene lists. Nucleic Acids Res..

[148] McHugh ML (2012). Interrater reliability: the kappa statistic. Biochem. Med..

[149] Bursac Z, Gauss CH, Williams DK, Hosmer DW (2008). Purposeful selection of variables in logistic regression. Source Code Biol. Med..

[150] Granados-Soler, J. L. *Molecular Characterisation of Feline Mammary Tumours* Ph.D thesis, Tierärztliche Hochschule Hannover, (2019).

