# Publisher Correction: Analysis of Copy-Number Variations and Feline Mammary Carcinoma Survival

**DOI:** 10.1038/s41598-020-62222-5

**Published:** 2020-03-20

**Authors:** José Luis Granados-Soler, Kirsten Bornemann-Kolatzki, Julia Beck, Bertram Brenig, Ekkehard Schütz, Daniela Betz, Johannes Junginger, Marion Hewicker-Trautwein, Hugo Murua Escobar, Ingo Nolte

**Affiliations:** 1 Small Animal Clinic, University of Veterinary Medicine Hannover Foundation, Hannover, Germany; 2 Chronix Biomedical, Göttingen, Germany; 3 Institute of Veterinary Medicine, University of Göttingen, Göttingen, Germany; 4 Department of Pathology, University of Veterinary Medicine Hannover Foundation, Hannover, Germany; 5 Haematology, Oncology and Palliative Medicine, Clinic III, University of Rostock, Rostock, Germany

Correction to: *Scientific Reports* 10.1038/s41598-020-57942-7, published online 22 January 2020

In the original version of this Article, the author Hugo Murua Escobar was incorrectly indexed. This error has now been corrected.

