# Supplementary material for: Analysis of Copy-Number Variations and Feline Mammary Carcinoma Survival
Source: Sci Rep. 2020 Jan 22;10:1003. doi: 10.1038/s41598-020-57942-7 (PMC6976565; doi:10.1038/s41598-020-57942-7)
Supplement: Supplementary file 1 — Supporting Information. [file 41598_2020_57942_MOESM1_ESM.pdf]

Supplementary material

Analysis of Copy-Number Variations and Feline Mammary Carcinoma Survival

José Luis Granados-Soler, Kirsten Bornemann-Kolatzki, Julia Beck, Bertram Brenig, Ekkehard Schütz, Daniela Betz, Johannes Junginger, Marion Hewicker-Trautwein, Hugo Murua-Escobar, Ingo Nolte

Supplementary Table 1. Characteristics of cases included in this study.

Clinical feature	Group TC (n=25)	Group SC (n=8)
Age		
< 10 years	5 (20%)	2 (25%)
> 10 years	20 (80%)	6 (75%)
Breed		
Domestic shorthair	19 (76%)	4 (50%)
Siamese	0 (0%)	1(12.5%)
Persian	1 (4 %)	0 (0%)
Norwegian Forest cat	2 (8%)	1 (12.5%)
Chartreux	0 (0%)	2 (25%)
Abyssinian	1 (4 %)	0 (0%)
British Shorthair	1 (4 %)	0 (0%)
Maine Coon	1 (4 %)	0 (0%)
Neutered		
No	12 (48%)	5 (62.5%)
Yes	13 (52%)	3 (37.5%)
Tumour size [1]		
<2 cm	10 (40%)	2 (25%)
2-3 cm	8 (32%)	4 (50%)
>3 cm	7 (28%)	2 (25%)
Lymph-node invasion		
Negative	17 (68%)	1 (12.5%)
Positive	8 (32%)	7 (87.5%)
Clinical stage [1]		
I	9 (36%)	1 (12.5%)
II	4 (16%)	0 (0%)
III	12 (48%)	7 (87.5%)
IV	0 (0%)	0 (0%)
TC, tubulopapillary carcinomas; SC, solid carcinomas and comedocarcinomas.		

Supplementary Table 2. Immunohistochemical features and molecular subtyping of cases included in this study.

Tumour feature	Group TC (n=25)	Group SC (n=8)
ER status [2-4]		
Negative	14 (56%)	8 (100%)
Positive	10 (40%)	0 (0%)
Unknown	1 (4%)	0 (0%)
PR status [2-4]		
Negative	12 (48%)	8 (100%)
Positive	11 (44%)	0 (0%)

Unknown	2 (8%)	0 (0%)
***f*HER2 score** [4-7]		
0 (negative)	5 (20%)	2 (25%)
1+ (negative)	14 (56%)	5 (62.5%)
2+ (equivocal)	4 (16%)	1 (12.5%)
3+ (positive)	2 (8%)	0 (0%)
***f*HER2 CNGs (Log2 ≥ 0.5)**		
Absent	24 (96%)	5 (62.5%)
Present	1 (4%)	3 (37.5%)
***f*HER2 status after HER2-associated CNGs detection**		
Negative	25 (100%)	7 (87.5%)
Positive	0(0%)	1 (12.5%)
CK5/6 status [8]		
Negative	19 (76%)	5 (62.5%)
Positive	6 (24%)	3 (37.5%)
Ki-67 index [9]		
Low	11 (44%)	0 (0%)
High	14 (66%)	8 (100%)
Molecular subtype		
LA	10 (40%)	0 (0%)
LB *f*HER2+	2 (8%)	0 (0%)
LB *f*HER2-	5 (20%)	0 (0%)
*f*HER2+	0 (0%)	1 (12.5%)
Normal-like TN	4 (16%)	4 (50%)
Basal-like TN	2 (8%)	3 (37.5%)
Unknown	2 (8%)	0 (0%)

TC, tubulopapillary carcinomas; SC, solid carcinomas and comedocarcinomas; LA, luminal A; LB *f*HER2-, luminal B *f*HER2 negative; LB *f*HER2+, luminal B *f*HER2 positive, *f*HER2+, *f*HER2 positive; and TN, triple-negative. *f*HER2 CNGs: FCA E1 38–44 Mb

Supplementary Table 3. Histological grading and Ki-67 percent agreement calculation.

Histopathological features	Group TC (n=25)	Group SC (n=8)	Low ki-67	High ki-67
EE grading system				
I	12 (48%)	0 (0%)	4 (33.3%)	8 (66.7%)
II	9 (36%)	2 (25%)	2 (18.8%)	9 (81.2%)
III	4 (16%)	6 (75%)	1 (10%)	9 (90%)
MMEE grading system				
I	20 (80%)	1 (12.5%)	10 (47.6%)	11 (52.4%)
II	3 (12%)	6 (75%)	1 (18.1%)	8 (88.9%)
III	2 (8%)	1 (12.5%)	0 (0%)	3 (100%)
Mills-2015 grading system				
I	6 (24%)	0 (0%)	4 (66.7%)	2 (33.3%)
II	12 (48%)	4 (50%)	7 (43.7%)	9 (56.3)
III	7 (28%)	4 (50%)	0 (0%)	11 (100%)

TC, tubulopapillary carcinomas; SC, solid carcinomas and comedocarcinomas; EE, Elston and Ellis [10,11]; MMEE, mitotic-modified EE [12,13]; Mills-2015, novel histological malignancy grading system for evaluation of FMCs [12,13]. I: well-differentiated, II: moderately-differentiated, and III: poorly-differentiated. Ki-67 index, low <14%, and high ≥14% [9].

Supplementary Table 4. Follow-up.

Variable	Group TC (n=25)	Group SC (n=8)
Local recurrence		
No	13 (52%)	3 (37.5%)
Yes	12 (48%)	5 (62.5%)
Distant metastases		
No	16 (64%)	8 (100%)

Yes	9 (36%)	0 (0%)
Tumour-related death		
No	14 (56%)	0 (0.0%)
Yes	11 (44%)	8 (100.0%)
Alive at the end of the study period		
No	17 (68%)	8 (100.0%)
Yes	8 (32%)	0 (0.0%)
TC, tubulopapillary carcinomas; and SC, solid carcinomas and comedocarcinomas.		

Supplementary table 5. Mapped reads per sample.

Group	Molecular subtype	Reads	Mapped reads	Mapped reads (%)	Aberrant bins (%) CNV-seq [14]	Aberrant bins (%) CNVKit [15]	Overlapped aberrant bins (%)*	CNVs score**
TC	LA	35,146,002	31,128,503	88.6	0.98	1.96	0.98	low
TC	LA	36,855,858	29,863,489	81.0	0.29	12.8	0.22	low
TC	LA	32,886,396	25,911,136	78.8	51.7	6.7	6.64	low
TC	LA	39,308,826	33,964,394	86.4	1.91	4.48	0.83	low
TC	LA	29,492,146	24,591,489	83.4	4.19	3.0	1.13	low
TC	LA	42,174,474	36,985,824	87.7	8.84	16.9	8.52	low
TC	LB fHER2+	30,065,412	25,633,485	85.3	25.0	73.2	24.6	high
TC	LB fHER2+	47,942,522	37,854,575	79.0	3.99	37.6	2.3	low
TC	LB *f*HER2-	46,425,624	35,236,683	75.9	59.1	59.6	51.6	high
TC	LB *f*HER2-	38,429,936	32,241,912	83.9	40.8	61.4	26.4	high
TC	LB *f*HER2-	36,588,186	31,951,371	87.3	7.05	11.4	5.34	low
TC	LB *f*HER2-	38,354,266	32,814,356	85.6	8.55	4.38	2.51	low
TC	normal-like TN	22,835,770	19,821,655	86.8	20.1	13.8	12.3	high
TC	normal-like TN	44,214,848	34,567,176	78.2	12.1	4.1	2.6	low
TC	normal-like TN	33,158,036	27,588,258	83.2	40.3	29.0	20.8	high
TC	basal-like TN	33,798,816	28,525,657	84.4	11.9	11.9	11.9	high
TC	not-classified	34,333,294	30,368,311	88.5	50.3	52.4	41.4	high
TC	not-classified	36,762,578	7,451,815	20.3	33.4	15.9	1.82	low
SC	*f*HER2+	42,300,368	35,296,722	83.4	63.3	14.5	8.68	high
SC	normal-like TN	37,039,032	31,018,359	83.7	19.8	4.99	4.05	low
SC	normal-like TN	33,695,620	28,995,733	86.1	38.5	92.7	33.6	high
SC	normal-like TN	39,990,342	32,154,081	80.4	68.9	78.4	60.6	high
SC	normal-like TN	32,310,280	27,695,454	85.7	49.7	9.1	6.68	low
SC	basal-like TN	39,039,402	34,882,641	89.4	33.7	62.5	28.1	high
SC	basal-like TN	36,570,098	25,990,743	71.1	73.3	93.5	68.8	high
SC	basal-like TN	28,468,460	25,620,723	90.0	53.6	55.4	25.7	high
Control***	-	31,330,660	27,918,236	89.1	0	0	0	-
Control***	-	36,449,280	31,715,188	87.0	0	0	0	-

*Validated using both algorithms (CNVKit [15] and CNV-seq [14]), **high-CNVs (≥median overlapped aberrant genomic windows), and low-CNVs (<median overlapped aberrant genomic windows); data calculated from CNVKit [15] and CNV-seq [14], ***healthy feline mammary frozen tissue. TC, tubulopapillary carcinomas; and SC, solid carcinomas and comedocarcinomas; LA, luminal A; LB *f*HER2+, luminal B/*f*HER2-positive; LB *f*HER2-, luminal B/*f*HER2-negative; *f*HER2+, *f*HER2-positive; and TN, triple-negative.

Supplementary Table 6. Validated CNVs detected in this study.

CNV ID	FCA	Start (bp)	End (bp)	Affected animals										
				Type of aberration		Histological group		Molecular subtype						
				CNLs	CNGs	TC n=18	SC n=8	LA n=6	LB *f*HER2+ n=2	LB *f*HER2- n=4	*f*HER2+ n=1	Normal-like TN n=7	Basal-like TN n=4	Not-classified n=2
1	A1	1,000,001	28,000,001	2	1	0	3	0	0	0	0	1	2	0
2	A1	28,000,001	30,000,001	3	1	1	3	0	1	0	0	1	2	0
3	A1	31,000,001	37,572,997	3	1	3	1	0	1	0	0	0	2	1
4	A1	39,323,189	65,000,001	5	2	4	3	1	1	0	1	1	2	1
5	A1	65,000,001	66,000,001	2	1	2	1	1	1	0	0	1	0	0
6	A1	66,000,001	85,056,009	2	1	0	3	0	0	0	0	1	2	0
7[*]	A1	85,056,009	105,000,001	3	0	0	3	0	0	0	0	0	3	0
8[*]	A1	105,000,001	124,000,001	5	0	1	4	0	0	0	0	2	3	0
9	A1	126,385,682	149,000,001	4	1	3	2	0	1	0	0	2	1	1
10	A1	167,113,735	179,000,001	4	1	3	2	1	1	0	0	1	1	1
11[***,†]	A1	179,000,001	194,000,001	6	2	5	3	1	2	0	0	3	1	1
12	A1	198,000,001	203,729,247	5	2	5	2	1	2	0	0	2	1	1
13	A1	209,000,001	216,000,001	2	2	2	2	0	0	0	0	2	1	1
14	A1	221,000,001	238,285,818	2	2	2	2	0	0	0	0	2	1	1
15	A2	1,836,083	19,348,854	5	1	4	2	0	0	1	0	2	2	1
16[*,†,‡]	A2	23,000,001	37,305,285	8	0	3	5	0	1	2	0	2	3	0
17[*,†]	A2	37,305,285	48,000,001	7	0	3	4	0	1	2	0	2	2	0
18[*,†]	A2	48,000,001	52,000,001	6	0	3	3	0	1	2	0	2	1	0
19[*]	A2	53,000,001	73,000,001	4	0	2	2	0	0	2	0	1	1	0
20[*]	A2	73,000,001	92,394,961	5	0	2	3	0	0	2	0	1	2	0
21	A2	94,453,859	130,000,001	4	1	3	2	0	1	1	0	2	1	0
22[*]	A2	130,000,001	144,000,001	4	0	2	2	0	1	1	0	1	1	0
23	A2	145,000,001	167,820,472	4	1	2	3	0	0	1	0	3	1	0
24[**,†,‡]	A3	1,000,001	31,000,001	0	8	3	4	0	0	2	0	3	2	1
25[**]	A3	54,248,782	71,000,001	0	2	1	1	0	0	0	0	2	0	0
26[**]	A3	71,000,001	90,000,001	0	2	0	2	0	0	0	0	2	0	0
27[**]	A3	92,308,192	120,000,001	0	3	1	2	0	0	0	0	3	0	0
28[**]	A3	120,000,001	141,415,341	0	5	1	4	0	0	0	0	3	2	0
29[†]	B1	1,000,001	23,000,001	7	1	6	2	1	1	2	0	2	1	1
30[†]	B1	23,000,001	31,000,001	6	1	6	1	1	1	2	0	2	0	1
31	B1	31,000,001	48,814,379	5	1	5	1	1	1	2	0	1	0	1
32[*]	B1	48,814,379	50,000,001	2	0	1	1	1	0	0	0	0	1	0
33[*]	B1	51,000,001	183,000,001	4	0	2	2	0	0	2	0	1	1	0
34[*,†]	B1	184,000,001	204,316,084	7	0	4	3	1	0	2	0	1	2	1
35	B2	1,000,001	24,000,001	3	1	2	2	0	0	1	0	2	1	0
36	B2	24,000,001	46,833,593	2	1	1	2	0	0	1	0	1	1	0
37	B2	49,000,001	118,000,001	4	1	2	3	0	1	0	0	1	3	0
38	B2	120,291,416	135,000,001	2	1	1	2	0	0	1	0	1	1	0
39[**]	B2	135,000,001	154,226,162	0	1	1	0	0	0	1	0	0	0	0
40[*]	B3	1,036,052	21,000,001	1	0	0	1	0	0	0	0	1	0	0
41[*]	B3	21,000,001	41,000,001	1	0	0	1	0	0	0	0	0	1	0
42[*]	B3	41,000,001	60,000,001	2	0	0	2	0	0	0	0	1	1	0
43	B3	61,510,334	122,000,001	2	1	1	2	0	0	1	0	1	1	0
44	B3	122,000,001	148,403,665	1	1	2	0	0	0	1	0	0	0	1
45[**,†,‡]	B4	1,000,001	29,000,001	0	9	3	6	0	1	1	1	3	3	0
46[**,†]	B4	29,000,001	33,000,001	0	7	3	4	0	1	1	1	3	1	0
47[**,†,‡]	B4	33,000,001	58,335,105	0	8	4	4	0	1	1	1	3	1	1
48[**]	B4	60,219,879	107,196,991	0	5	3	2	0	0	1	0	3	0	1
49	B4	112,459,969	124,000,001	1	3	3	1	0	0	2	0	1	0	1
50[†]	B4	126,000,001	143,245,922	1	6	2	5	0	0	1	1	2	2	1
51[*]	C1	2,770,047	21,000,001	4	0	2	2	0	0	1	0	1	1	1

52†	C1	21,000,001	45,000,001	6	1	3	4	1	0	1	0	2	2	1
53*	C1	47,201,234	90,000,001	5	0	2	3	0	1	1	0	1	2	0
54*	C1	92,000,001	120,000,001	5	0	1	4	0	0	1	0	2	2	0
55*	C1	122,000,001	200,000,001	5	0	2	3	0	1	1	0	1	2	0
56	C1	203,000,001	220,507,052	3	1	3	1	0	0	1	0	0	2	1
57**	C2	1,000,001	20,000,001	0	5	2	3	0	0	1	0	1	2	1
58	C2	21,000,001	48,000,001	1	3	2	2	0	0	0	0	0	3	1
59**	C2	50,331,871	120,000,001	0	1	1	0	0	0	0	0	0	0	1
60**	C2	139,000,001	157,565,780	0	5	1	4	0	0	0	0	1	3	1
61†	D1	1,111,220	20,000,001	6	1	3	4	0	0	2	0	3	2	0
62*,†,‡	D1	21,000,001	39,375,734	8	0	3	5	0	0	2	0	2	4	0
63*,†	D1	41,227,239	97,000,001	6	0	2	4	0	0	2	0	1	3	0
64*	D1	98,000,001	112,284,005	3	0	2	1	0	0	2	0	0	1	0
65*	D1	112,284,005	117,000,001	1	0	1	0	0	0	1	0	0	0	0
66	D2	1,000,001	20,000,001	5	1	3	3	1	1	1	1	0	2	0
67	D2	21,000,001	33,000,001	2	1	1	2	0	1	0	0	0	2	0
68	D2	33,000,001	51,880,556	3	1	1	3	0	1	0	0	1	2	0
69*	D2	51,880,556	54,000,001	1	0	0	1	0	0	0	0	0	1	0
70	D2	54,000,001	88,941,831	4	3	3	4	0	1	0	0	2	3	1
71	D3	1,000,001	35,000,001	3	1	3	1	0	0	2	0	1	0	1
72	D3	37,882,964	94,777,426	2	1	3	0	0	0	2	0	0	0	1
73†	D4	1,000,001	16,748,051	3	6	5	4	1	0	2	0	2	3	1
74	D4	18,684,934	37,000,001	3	2	3	2	1	0	0	0	2	1	1
75*	D4	37,000,001	58,000,001	2	0	1	1	1	0	0	0	1	0	0
76	D4	58,000,001	73,000,001	4	1	3	2	1	0	1	0	1	1	1
77	D4	75,000,001	93,149,910	4	1	3	2	1	0	1	0	0	2	1
78†	E1	1,000,001	17,000,001	1	6	4	3	1	0	1	1	2	1	1
79**,†	E1	19,346,552	37,000,001	0	7	3	4	0	0	1	1	3	1	1
80**,†	E1	38,000,001	44,000,001	0	7	3	4	0	0	1	1	3	1	1
81**,†	E1	44,000,001	62,997,655	0	7	3	4	0	0	1	1	3	1	1
82	E2	1,000,001	17,872,790	2	1	2	1	1	0	0	0	0	1	1
83	E2	20,021,674	45,000,001	2	2	2	2	0	0	0	0	2	1	1
84*	E2	45,000,001	63,365,623	2	0	1	1	0	0	0	0	0	1	1
85†,‡,#	E3	1,111,912	34,464,880	1	10	6	5	0	1	2	1	3	3	1
86	E3	37,000,001	42,512,992	4	5	6	3	2	0	1	0	3	2	1
87**	F1	1,000,001	23,000,001	0	3	0	3	0	0	0	0	2	1	0
88**	F1	25,771,186	50,000,001	0	1	0	1	0	0	0	0	1	0	0
89**	F1	50,000,001	68,144,453	0	1	1	0	0	0	1	0	0	0	0
90**	F2	1,000,001	16,381,563	0	3	2	1	0	0	0	0	1	1	1
91**	F2	18,000,001	63,000,001	0	3	3	0	0	0	1	0	1	0	1
92**,†,‡	F2	64,000,001	82,358,132	0	8	5	3	0	0	2	0	2	2	2
93*,†,‡	X	1,000,001	26,000,001	9	0	4	5	1	0	1	0	5	2	0
94*,†	X	27,820,134	45,240,957	7	0	4	3	1	0	1	0	3	2	0
95	X	45,668,770	85,504,303	3	1	4	0	1	0	2	0	0	1	0
96*,†	X	86,588,413	108,000,001	7	0	3	5	1	0	1	0	3	3	0
97*,†,‡	X	108,000,001	126,277,513	10	0	4	6	2	0	1	0	4	3	0

FCA, feline chromosome; CNL, copy-number loss; and CNG, copy-number gain, *regions exclusively affected by CNLs (affected regions=29), **region exclusively affected by CNGs (affected regions=22), †CNVs present in >20% of evaluated patients (affected regions=51), ‡CNVs present in >30% of evaluated patients (affected regions=14), #CNVs present in >40% of evaluated patients (affected regions=1).

Supplementary Table 7. CNVs negatively influencing DFS and/or OS in the univariate analysis.

Region ID	FCA	Type of aberration	Start (bp)	End (bp)	DFS *p*-value	Cancer-related OS *p*-value
16*,†,‡	A2	CNL	23,000,001	37,305,285	0.009	0.01
17*,†	A2	CNL	37,305,285	48,000,001	0.04	0.05

24[**,†,‡]	A3	CNG	1,000,001	31,000,001	0.003	0.002
29[†]	B1	CNL	1,000,001	23,000,001	0.00008	0.0003
45[**,†,‡]	B4	CNG	1,000,001	29,000,001	0.001	0.01
47[**,†,‡]	B4	CNG	33,000,001	58,335,105	0.05	NS
50[†]	B4	CNG	126,000,001	143,245,922	0.009	NS
52[†]	C1	CNL	21,000,001	45,000,001	0.04	NS
61[†]	D1	CNL	1,111,220	20,000,001	0.006	0.02
62[*,†,‡]	D1	CNL	21,000,001	39,375,734	0.00005	0.0004
63[*,†]	D1	CNL	41,227,239	97,000,001	0.001	0.004
73[†]	D4	CNG	1,000,001	16,748,051	0.009	0.006
79[**,†]	E1	CNG	19,346,552	37,000,001	0.05	NS
80[**,†]	E1	CNG	38,000,001	44,000,001	0.05	NS
81[**,†]	E1	CNG	44,000,001	62,997,655	0.05	NS
85[†,‡,#]	E3	CNG	1,111,912	34,464,880	0.04	NS
92[**,†,‡]	F2	CNG	64,000,001	82,358,132	0.0002	0.006

FCA, feline chromosome; CNL, copy-number loss; and CNG, copy-number gain, [*]regions exclusively affected by CNLs (affected regions=9), [**]region exclusively affected by CNGs (affected regions=10), [†] CNVs present in >30% of evaluated patients (affected regions=10), [‡] CNVs present in >40% of evaluated patients (affected regions=1).

Supplementary Table 8. Antibodies and corresponding negative and positive controls used in this study.

Antibody	Type	Clone	Company	Negative control	Positive control
ER	mouse anti-human	6F11	AbD Serotec	IgG1	feline normal mammary gland
PR	mouse anti-human	hPRa 2	Invitrogen	IgG2a	feline normal mammary gland
HER2	mouse anti-human	CB11	Novocastra	IgG1	human mammary tissue and pellets from cell lines<br>overexpressing HER-2*, feline mammary carcinoma
CK5/6	mouse anti-human	D5/16B4	Dako	IgG1	feline skin
Ki-67	mouse anti-human	MIB-1	Dako	IgG1	feline normal small intestine

ER, oestrogen receptor; PR, progesterone receptor; HER2, Human epidermal growth factor receptor; CK5/6, cytokeratin 5 and 6; and Ki-67, marker of proliferation Ki-67, *kindly provided by Prof, Dr, H,-H, Kreipe, Department of Pathology, Hannover Medical School.

Supplementary Table 9. Immunohistochemical-based St. Gallen classification for FMCs.

Subtype	ER and PR status	*f*HER2	CK5/6	Ki-67 index
LA	ER+ and/or PR+	-	any	low
LB *f*HER2-	ER+ and/or PR+	-	any	high
LB *f*HER2+	ER+ and/or PR+	+	any	any
*f*HER2+	ER- and PR-	+	any	any
Normal-like TN	ER- and PR-	-	-	any
Basal-like TN	ER- and PR-	-	+	any

-negative; +positive; high ≥14%; low <14%.

Supplementary Table 10. Allred score guidelines for ER and PR staining.

Percentage of positive tumour cells	Score for percentage of positive tumour cells
0	0
< 1	1

1–10	2
10–33	3
33–66	4
>66	5
**Intensity**	**Score for average intensity of staining**
none	0
weak	1
moderate	2
strong	3
The Allred score (0–8) equals de ∑ of both scores, Adapted from [2-4].	

Supplementary Table 11. *f*HER2 IHC scoring criteria.

Description	Score	Score for St, Gallen classification
No staining	0	-
Weak, incomplete membranous staining in any proportion of tumour cells	+1	-
Complete membrane staining that is either no uniform or weak in intensity but with obvious circumferential distribution in at least 10% of cells	+2	equivocal
Uniform intense membrane staining of at least 10% of invasive tumour cells	+3	+
–negative, and +positive, Adapted from [4-7,16]		

References

1 McNeill, C. J. *et al.* Evaluation of adjuvant doxorubicin-based chemotherapy for the treatment of feline mammary carcinoma. *J Vet Intern Med* **23**, 123-129, doi:10.1111/j.1939-1676.2008.0244.x (2009).

2 Mohsin, S. K. *et al.* Progesterone receptor by immunohistochemistry and clinical outcome in breast cancer: a validation study. *Mod Pathol* **17**, 1545-1554, doi:10.1038/modpathol.3800229 (2004).

3 Soares, M., Correia, J., Murta, A. & Ferreira, F. Immunophenotyping of primary and metastatic lesions in feline mammary tumors - are they equal? *Microscopy and Microanalysis* **19**, 19-20, doi:10.1017/s1431927613000718 (2013).

4 Soares, M. *et al.* Molecular based subtyping of feline mammary carcinomas and clinicopathological characterization. *Breast* **27**, 44-51, doi:10.1016/j.breast.2016.02.016 (2016).

5 Park, S. *et al.* Characteristics and outcomes according to molecular subtypes of breast cancer as classified by a panel of four biomarkers using immunohistochemistry. *Breast* **21**, 50-57, doi:10.1016/j.breast.2011.07.008 (2012).

6 Soares, M. *et al.* Feline HER2 protein expression levels and gene status in feline mammary carcinoma: optimization of immunohistochemistry (IHC) and in situ hybridization (ISH) techniques. *Microsc Microanal* **19**, 876-882, doi:10.1017/S1431927613001529 (2013).

7 Wolff, A. C. *et al.* Human Epidermal Growth Factor Receptor 2 Testing in Breast Cancer: American Society of Clinical Oncology/College of American Pathologists Clinical Practice Guideline Focused Update. *Arch Pathol Lab Med* **142**, 1364-1382, doi:10.5858/arpa.2018-0902-SA (2018).

8 Brunetti, B. *et al.* Molecular phenotype in mammary tumours of queens: correlation between primary tumour and lymph node metastasis. *J Comp Pathol* **148**, 206-213, doi:10.1016/j.jcpa.2012.05.012 (2013).

9 Soares, M. *et al.* Ki-67 as a Prognostic Factor in Feline Mammary Carcinoma: What Is the Optimal Cutoff Value? *Veterinary Pathology* **53**, 37-43, doi:10.1177/0300985815588606 (2016).

10 Elston, C. W. & Ellis, I. O. Pathological prognostic factors in breast cancer. I. The value of histological grade in breast cancer: experience from a large study with long-term follow-up. *Histopathology* **19**, 403-410, doi:10.1111/j.1365-2559.1991.tb00229.x (1991).

11 Castagnaro, M. *et al.* Tumour grading and the one-year post-surgical prognosis in feline mammary carcinomas. *J Comp Pathol* **119**, 263-275, doi:10.1016/s0021-9975(98)80049-2 (1998).
12 Mills, S. W. *et al.* Prognostic value of histologic grading for feline mammary carcinoma: a retrospective survival analysis. *Vet Pathol* **52**, 238-249, doi:10.1177/0300985814543198 (2015).
13 Dagher, E., Abadie, J., Loussouarn, D., Campone, M. & Nguyen, F. Feline Invasive Mammary Carcinomas: Prognostic Value of Histological Grading. *Vet Pathol* **56**, 660-670, doi:10.1177/0300985819846870 (2019).
14 Xie, C. & Tammi, M. T. CNV-seq, a new method to detect copy number variation using high-throughput sequencing. *BMC Bioinformatics* **10**, 80, doi:10.1186/1471-2105-10-80 (2009).
15 Talevich, E., Shain, A. H., Botton, T. & Bastian, B. C. CNVkit: Genome-Wide Copy Number Detection and Visualization from Targeted DNA Sequencing. *PLoS Comput Biol* **12**, e1004873, doi:10.1371/journal.pcbi.1004873 (2016).
16 Dako. Herceptest™ Interpretation Manual-Breast Cancer. 1-52 (2014).
